# Overexpression of a Plasma Membrane Bound Na^+^/H^+^ Antiporter-Like Protein (*SbNHXLP*) Confers Salt Tolerance and Improves Fruit Yield in Tomato by Maintaining Ion Homeostasis

**DOI:** 10.3389/fpls.2016.02027

**Published:** 2017-01-06

**Authors:** P. Hima Kumari, S. Anil Kumar, Pramod Sivan, Ramesh Katam, Prashanth Suravajhala, K. S. Rao, Rajeev K. Varshney, Polavarapu B. Kavi Kishor

**Affiliations:** ^1^Department of Genetics, Osmania UniversityHyderabad, India; ^2^Department of Biosciences, Sardar Patel UniversityAnand, India; ^3^Department of Biological Sciences, College of Science and Technology, Florida A&M UniversityTallahassee, FL, USA; ^4^Bioclues OrganizationHyderabad, India; ^5^Center of Excellence in Genomics, International Crops Research Institute for the Semi-Arid TropicsHyderabad, India

**Keywords:** *SbNHXLP*, tomato, salt stress, co-immunoprecipitation, *CHX2*

## Abstract

A Na^+^/H^+^ antiporter-like protein (NHXLP) was isolated from *Sorghum bicolor* L. (*SbNHXLP*) and validated by overexpressing in tomato for salt tolerance. Homozygous T_2_ transgenic lines when evaluated for salt tolerance, accumulated low Na^+^ and displayed enhanced salt tolerance compared to wild-type plants (WT). This is consistent with the amiloride binding assay of the protein. Transgenics exhibited higher accumulation of proline, K^+^, Ca^2+^, improved cambial conductivity, higher PSII, and antioxidative enzyme activities than WT. Fluorescence imaging results revealed lower Na^+^ and higher Ca^2+^ levels in transgenic roots. Co-immunoprecipitation experiments demonstrate that *Sb*NHXLP interacts with a *Solanum lycopersicum* cation proton antiporter protein2 (*Sl*CHX2). qRT-PCR results showed upregulation of *SbNHXLP* and *SlCHX2* upon treatment with 200 mM NaCl and 100 mM potassium nitrate. *SlCHX2* is known to be involved in K^+^ acquisition, and the interaction between these two proteins might help to accumulate more K^+^ ions, and thus maintain ion homeostasis. These results strongly suggest that plasma membrane bound *Sb*NHXLP involves in Na^+^ exclusion, maintains ion homeostasis in transgenics in comparison with WT and alleviates NaCl stress.

## Introduction

Salt stress limits plant growth and productivity by ion toxicity and water uptake by decreasing the water potential (Munns and Tester, 2008). To combat the problem of ion toxicity, plants have developed a number of strategies that are crucial for their survival. Plants sense the salt stress through signal transduction and respond by modulating biochemical, physiological, and molecular activities (Zhu, 2002). Plants prevent accumulation of Na^+^ in the cytosol to overcome Na^+^ toxicity either by Na^+^ efflux or compartmentalization of it into vacuoles through sodium-proton antiporters (NHXs) which belong to the cation/proton antiporter (CPA1) family of transporters (Mäser et al., 2001). Eight *NHX* genes have been reported in eukaryotic systems except in yeast (contains single *NHX*) and in model plants like *Arabidopsis* and rice they have been characterized to some extent (Bassil et al., 2011a,b; Zhang et al., 2015). While Na^+^ exclusion is carried out at the plasma membrane *via* salt overly sensitive (SOS) pathway (Zhang et al., 2001; Zhu, 2003), its sequestration is performed by NHX that is located at the tonoplast. Na^+^ efflux from plasma membrane to the apoplast is achieved by *SOS1* gene, or NHX7, located at root epidermal cells of plants. Accumulation of Na^+^ inside the vacuoles reduces the toxic levels of Na^+^ in cytosol with concomitant increase in the vacuolar osmotic potential generating more negative water potential, which favors uptake of water into the cells (Blumwald, 2000). To transport Na^+^ or K^+^ across the membranes, plant transporters utilize proton electrochemical gradients (Blumwald, 2000; Serrano and Rodriguez-Navarro, 2001). Salinity causes ionic imbalance by decreasing K^+^ conductance through the AKT1 channel (Qi and Spalding, 2004). Usually, plants maintain high K^+^/Na^+^ ratio, but this ion ratio is disturbed during salt stress due to leakage of K^+^ from the root cells (Shabala, 2000; Chen et al., 2005). Both K^+^ and Na^+^ are exchanged for H^+^ in plants, and the exchange is mediated by NHXs (Sanders, 2000; Zhu, 2003). NHX transporters are associated with a wide variety of functions including maintenance of K^+^ homeostasis (Leidi et al., 2010), long-distance transport of Na^+^ from root to shoot (Shi et al., 2002; Wu et al., 2016), salt tolerance (Zhang and Blumwald, 2001; Bassil et al., 2011b), flower opening and petal coloration (Yoshida et al., 2009), protein targeting and trafficking (Bassil et al., 2011a), cell expansion and flower development (Bassil et al., 2011b), and stomatal functioning (Barragan et al., 2012). NHX multiprotein family members localized in vacuolar (NHX1-4), endosomal (NHX5-6), and plasma membranes (SOS1/NHX7 and NHX8) have been reported earlier (Shi et al., 2002; Pardo et al., 2006; Bassil et al., 2011a), but not NHX-like proteins.

*NHX1* overexpression improved salt tolerance in numerous species by sequestering Na^+^ into the vacuole (Apse et al., 1999; Zhang and Blumwald, 2001; Apse and Blumwald, 2007; Kronzucker and Britto, 2011). *Lycopersicum esculentum NHX2* (*LeNHX2*) knockdown in tomato (*Solanum lycopersicum* L.) and double knock out *atnhx5*/*atnhx6* in *Arabidopsis* displayed salt sensitivity implying that they are associated with salinity stress (Rodriguez-Rosales et al., 2008; Bassil et al., 2011a). Like-wise, *AtNHX5* gene conferred tolerance to Na^+^ in *Torenia* (Shi et al., 2008). *SOS1* gene overexpression improved salt tolerance in *Arabidopsis, Lycopersicum esculentum, Brassica napus*, and *Nicotiana tabacum* (Apse et al., 1999; Zhang and Blumwald, 2001; Shi et al., 2003; Yadav et al., 2012). Its overexpression has increased salt tolerance in transgenic tobacco by maintaining a higher K^+^/Na^+^ ratio (Yue et al., 2012). Tomato, an important vegetable crop suffers from salt stress. Genetic engineering aids in the transfer of candidate genes for the production of salt tolerant crops and for sustainable agriculture. Transgenic tomato plants overexpressing a vacuolar *NHX1* were able to grow, flower, and fruit in the presence of 200 mM NaCl (Zhang and Blumwald, 2001). Tomato with reduced *SOS1* expression resulted in the low accumulation of Na^+^ in stems than in the leaves indicating its role in partition of Na^+^ between plant organs (Olías et al., 2009). Further, *LeNHX* isoforms (*LeNHX1, LeNHX2, LeNHX3*, and *LeNHX4*) were observed in tomato with and without salt stress (Gálvez et al., 2012). Under salt stress, transgenic tomato overexpressing *LeNHX2* showed higher accumulation of K^+^ compared to wild-type plants (WT) (Huertas et al., 2013). Analysis of the cloned Na^+^-H^+^ antiporter revealed that its molecular weight matches with NHX2 proteins detected so far in various taxa, but localized to the plasma membrane like NHX7 and NHX8 proteins. Its molecular weight also did not match with NHX7/NHX8 proteins that have been identified till date and hence, it is named as NHX-like protein. Such *NHXLP* genes have not been cloned and validated for their function earlier in any plant species so far to the best of our knowledge. Its functional validation in tomato revealed low accumulation of Na^+^, but high accumulation of K^+^ in transgenics and thus conferred salt tolerance.

## Materials and methods

### *NHXLP* gene cloning from *S. bicolor* and *in silico* analysis

Full length *SbNHXLP* coding sequence was retrieved from *S. bicolor* genome sequence using GENSCAN software (Burge and Karlin, 1998, http://genes.mit.edu/GENSCAN.html). From the BLAST (Altschul et al., 1990) output, end to end primers were designed to amplify the full length gene. PCR reaction was performed using gene specific primers (forward primer 5′ATGGGGCTCGATTTGGGAGCT3′ and reverse primer 5′TCAACTATGCTCAGCCTCTGTCA3′) with *S. bicolor* variety BTX623 cDNA. The amplified product was cloned into pTZ57R/T vector and sequenced. The vector pCAMBIA1302 harboring *SbNHXLP* gene driven by CaMV35S promoter and NOS PolyA terminator was mobilized into *Agrobacterium tumefaciens* strain LBA4404 using freeze-thaw method. *SbNHXLP* gene was characterized for the number of exons, introns, and their length by Gene Structure Display Server (GSDS) tool (http://gsds.cbi.pku.edu.cn/). Motif search database was used to identify the sodium proton exchanger motifs (http://www.genome.jp/tools/motif/). Prediction of transmembrane helices in protein was carried out using TMHMM (Krogh et al., 2001). Motifs were identified using the MEME software (Bailey et al., 2009). Homology model of *Sb*NHXLP protein was created and amiloride was docked to it using SYBYL FlexX software (Rarey et al., 1996).

### Genetic transformation and molecular characterization

Twelve-day-old cotyledonary and hypocotyl explants of tomato variety Pusa Early Dwarf (PED) were used for transformation studies. Cotyledonary and hypocotyl explants were cultured on Murashige and Skoog's (MS) medium (1962) fortified with 2 mg/L of thidiazuron (TDZ) (Murashige and Skoog, 1962). To test antibiotic sensitivity, explants were cultured on regeneration medium with different concentrations of hygromycin (0–10 mg/L) and cefotaxime (0–300 mg/L) separately. *Agrobacterium* infected explants were co-cultivated in dark for 2-days, after incubation, explants were sub-cultured onto selection medium (MS medium with 2 mg/L TDZ, 3 mg/L hygromycin, and 300 mg/L cefotaxime). Well-rooted transformants were acclimatized and grown in green house. Genomic DNA was isolated from leaf tissues of WT and transgenic plants (Doyle and Doyle, 1990). Putative transgenics were confirmed by PCR amplification using *SbNHXLP* and *hptII* gene specific primers. For gene copy number, genomic DNA (20 μg each) was digested with HindIII and probed with *hptII* gene (Sambrook and Russell, 2001). Total RNA (200 ng) isolated from transgenics and WT was used for first strand cDNA synthesis. Transcript levels were confirmed by reverse transcriptase PCR (RT-PCR) using *SbNHXLP* gene specific primers.

### Segregation analysis in T_1_ and T_2_ transgenics and immunolocalization of *Sb*NHXLP protein

T_1_ seeds were germinated on MS basal medium supplemented with 8 mg/L hygromycin. After 10-days of germination, Mendelian inheritance was observed by calculating hygromycin resistant and sensitive seedlings. Similarly, T_2_ seedlings obtained from selfed T_1_ generation were cultured on MS basal medium supplemented with 8 mg/L hygromycin. Homozygous lines were used for subsequent studies. Eight micron sized cryotome sections of transgenic stem were taken from 10-day-old seedlings and fixed in 4% paraformaldehyde. Immunolocalization method was performed with the following steps: blocking, incubation with primary antibodies, washing, incubation with secondary antibody conjugate, washing and putative visualization step, washing, putative counterstaining, and mounting. They were incubated with polyclonal Na^+^/H^+^ antiporter antibodies (Agrisera, AS09 484) and conjugated with Alexafluor (Invitrogen, USA) secondary antibodies. Sections were analyzed under inverted confocal microscope (Leica Microsystems) at 578 nm.

### Assessment of transgenic lines for salt tolerance

To assess salt tolerance, 45-day-old homozygous T_2_ transgenic lines (T_2−1−3,_T_4−1−6_, T_5−1−1_, T_7−1−15_) along with WT were treated with 200 mM NaCl for 15 alternate days. After treatment, stress was relieved by rewatering to see the extent of recovery.

### Measurement of proline, antioxidant enzyme, and PSII activities under salt stress

Forty five-day-old homozygous T_2_ transgenic lines (T_2−1−3,_T_4−1−6_, T_5−1−1_, T_7−1−15_) along with WT were treated with 200 mM NaCl for 15 alternate days. After 15-days of salt treatment, proline was estimated (Bates et al., 1973), activities of superoxide dismutase (SOD) (Beauchamp and Fridovich, 1971), and catalase (Luck, 1974) were determined. Protein concentration was measured by the method of Bradford (1976). For chlorophyll fluorescence, same age-group plants were treated with 200 mM NaCl for 7 days. PSII activity was measured before and after salt treatment (Strasser et al., 1995), experiments were repeated thrice and each time two plants were taken.

### Amiloride (A known inhibitor of Na^+^/H^+^ exchanger activity) binding assay, sodium green, and calcium green indicators

To determine the action of amiloride on the activity of *Sb*NHXLP protein, 45-day-old transgenic and WT plants were treated with 1 mM amiloride for 2 h. To find out the Na^+^ and Ca^2+^ accumulations, tomato seedlings were grown for 9-days and treated with 200 mM NaCl for 2 h. Root sections (8 μm) were cut from the mature zone, incubated in microfuge tubes in 500 μl of 10 mM Sodium Green (S6901, Invitrogen) and Calcium Green (C3012, Invitrogen) solutions separately. After 1 h incubation, samples were observed under confocal microscope.

### Ion analysis in transgenics

For ion analysis, 45-day-old T_5−1−1_ and T_7−1−15_ transgenic lines and WT were treated with 200 mM NaCl for 3 consecutive days. Root, stem, leaf, and flower tissue samples from transgenics and WT were digested in 3 ml of 3:1 HNO_3_: H_2_O_2_ for 24 h. Ions (Na^+^, K^+^, Ca^2+^, and Cl^−^) were measured using the inductively coupled plasma optical emission spectrometer (ICP-OES, Optima 2000DV, Perkin Elmer). The experiments were repeated thrice and each time two plants were taken.

### Anatomical studies by measurement of fiber and vessel elements

One-month-old T_5−1−1_ transgenic and WT were treated with 200 mM NaCl stress for 3-days. Thereafter, stem and root samples were macerated to measure the length and width of fibers and vessel elements. Small matchstick size wood pieces were macerated in Jeffrey's fluid (Berlyn and Mikshe, 1976). Semi-thin sections (1–2 μm) were taken with a glass knife using Reichert OM U3 ultramicrotome. Stained sections were observed and photographed using a Leica DM 2000 microscope attached with a Cannon DC 150 digital camera. The length and width of vessel elements and fibers were measured with an ocular micrometer scale mounted in a research microscope. The number of cambial cell layers was counted from the transverse sections. The fiber wall thicknesses, radial extent of xylem, and vessel density were recorded from transverse sections using ocular micrometer scale. For each parameter, 100 readings were taken from randomly selected elements from six replicates.

### Effect of salt stress treatment on fruit yield in the transgenic lines

Fruit yield (number and weight of fruits per plant) was measured in T_5−1−1_ and T_7−1−15_ transgenic lines along with WT. Sixty-day-old plants were treated with 200 mm NaCl stress for 15 alternate days. After treatment, stress was relieved by rewatering. The fruit yield was measured on 75th day.

### Protein-protein interaction (PPI) by co-immunoprecipitation (Co-IP)

Co-IP was performed following manufacturer's instructions (Pierce, Co-IP kit, 26149; Thermo Scientific). Ten microliters (1 μg/μl) of anti-NHX polyclonal antibodies (Agrisera, AS07 207) were immobilized onto A/G agarose beads. Total protein was isolated from transgenic and WT plants. Leaf tissue (50 mg) was ground with liquid nitrogen and washed with 1X Dulbecco's PBS, followed by addition of 500 μl of immunoprecipitation lysis buffer with gentle shaking for 5 min. Tubes were centrifuged at 13,000 × g for 10 min for pelleting. Agarose resin slurry (80 μl) was added to the spin column and allowed to settle. Storage buffer was removed by spinning. To this, 100 μl of 1X coupling buffer was added and centrifuged to discard the flow through. Cell lysate was added to the column having resin and incubated at 4°C for 1 h with gentle shaking and centrifuged at 1000 × g for 1 min. Flow through was added to immobilized antibody. Immobilized antibody beads and the protein mixture were incubated overnight at 4°C, 40 μl of protein A sepharose was added and incubated further for 2–3 h at 4°C. The beads were collected by centrifugation at 100 × g for 3 min at 4°C, and then washed 5 times with ice-cold IP buffer. The protein fractions were eluted from the beads and the immunoprecipitated samples were analyzed by SDS-PAGE and subjected to trypsin in-gel digestion and purification using trifluoroacetic acid, 5% formic acid, and acetonitrile. Agarose resin supplied along with the kit was taken as a negative control. Peptides were loaded on a matrix for mass spectrometric (MS-MS) analysis using 4700 plus MALDI TOF-TOF proteomics analyzer (Applied Biosystems, USA). MS-MS analyzed best peptide masses were taken for MASCOT analysis to know the plausible *SbNHXLP* interactant, which was further sequenced to obtain a full length sequence.

### Gene expression analysis by quantitative real time (qRT)-PCR

To find out the expression levels of *SbNHXLP* and *SlCHX2*, 1-month-old T_5−1−1_ transgenic and WT plants were treated with 200 mM NaCl and 200 mM mannitol to induce salt and drought stresses, respectively, for 72 h. Same age old plants were also treated with 10 mM KCl and 100 mM KNO_3_ for 72 h. Root, stem, and leaves were taken for relative expression studies of *SbNHXLP* and *SlCHX2* genes using POWER SYBR Green PCR Master Mix (Applied Biosystems). *SbNHXLP, SlCHX2*, and β-actin gene specific primers were used as shown in Table S1. The expression levels of *SbNHXLP* and *SlCHX2* genes in various samples were normalized to β-actin. No template controls (NTC) were used in every experiment. Experiments were performed with three technical replicates for each biological duplicate. The comparative 2^−ΔΔCT^ method was used to calculate the relative quantities of each transcript in the samples (Schmittgen and Livak, 2008).

### Statistical analysis

All experiments were carried out thrice with five plants in each treatment (unless otherwise mentioned). Mean, standard error mean, and *t*-test values were calculated with the help of excel sheet and the graphs were plotted using GraphPad softwareV6.01 Version (http://www.graphpad.com/scientific-software/prism).

## Results

### *SbNHXLP* gene cloning and *in silico* analysis

**Figure 1:**
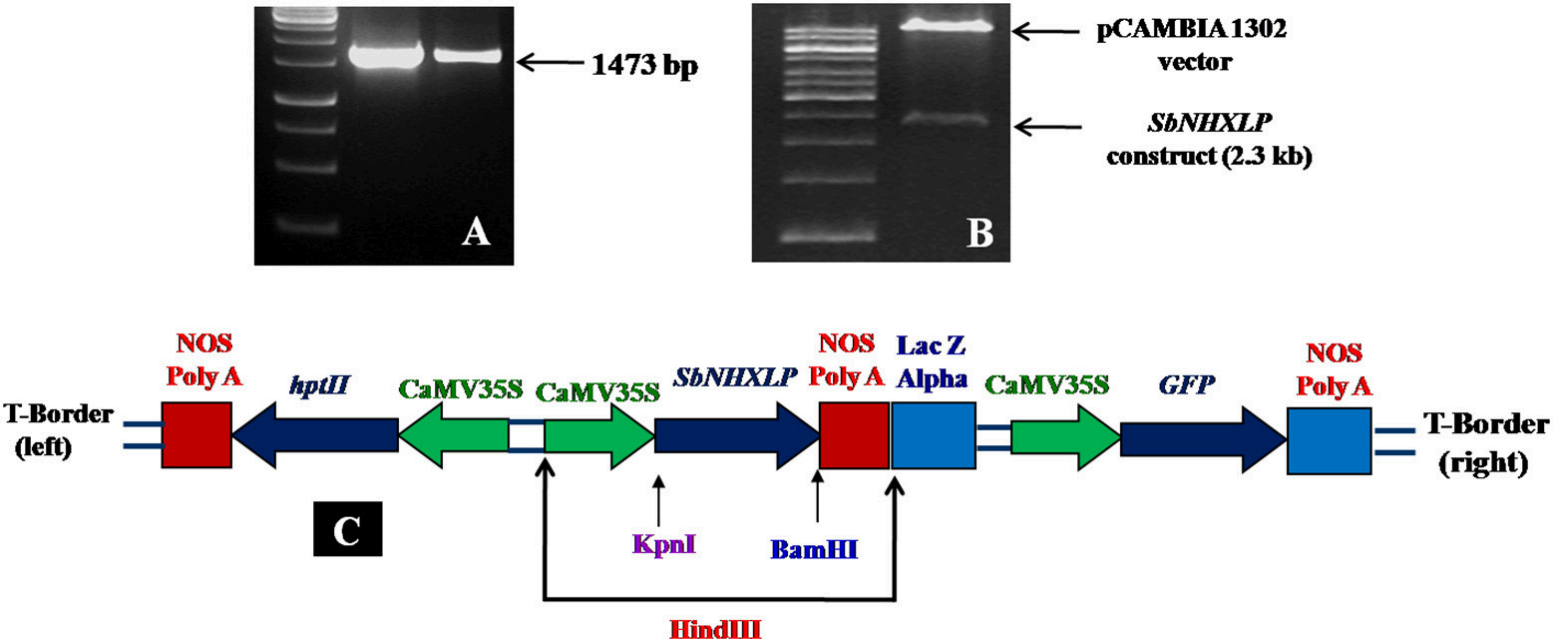
***SbNHXLP* gene isolation and construction. (A)** isolation of *SbNHXLP* (1473 bp), **(B)** gene construct and vector, **(C)** recombinant vector map of pCAMBIA1302-*SbNHXLP. SbNHXLP, Sorghum bicolor* Na^+^/H^+^ antiporter-like protein; *hptII*, hygromycin; CaMV35S, Cauliflower mosaic virus 35S promoter; nos, nopaline synthase terminator.

*SbNHXLP* full length cDNA (1473 bp) was amplified (Figure 1A) and deposited in NCBI (Accession number EU482408). *SbNHXLP* gene cassette driven by *CAMV35S* promoter and *NOS* terminator (Figure 1B) was cloned into pCAMBIA1302 vector using *HindIII* enzyme (Figure 1C). *In silico* analysis of *SbNHXLP* exhibited 93, 87, and 74% homology with NHX2, 83, 84, and 71% homology with NHX3 protein of maize, rice, and *Arabidopsis*, respectively. It has 12 exons and 11 introns (Figure S1A) and is localized on the chromosome number 9. Two Na^+^-H^+^ exchanger motifs were detected in *SbNHXLP* (Figure S1B). Further, *Sb*NHXLP revealed that it is a transmembrane protein (Figure S1C) which has eight motifs (Figure S1D) with varying number of amino acids as shown in Table S2. Though its molecular weight did not match with NHX7/NHX8 proteins, it is localized on the plasma membrane like that of NHX7 and NHX8 proteins which is later mirrored using immunolocalization. *Sb*NHXLP was modeled and a drug inhibitor amiloride was docked to the conserved domain LLFIYLLPPI, indicating that amiloride inhibits its activity (Figure S1E).

### Molecular characterization of transgenics

**Figure 2:**
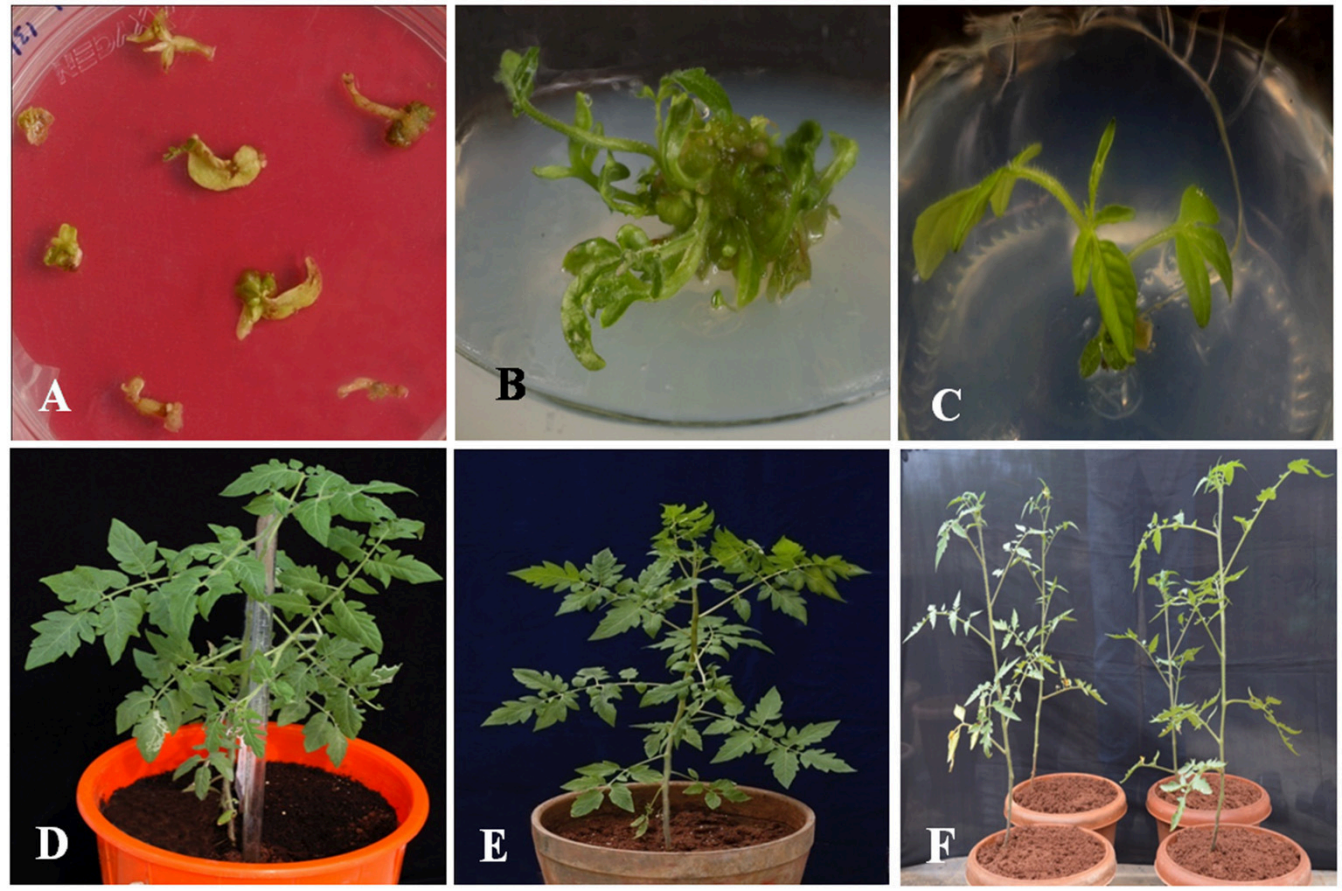
**Different developmental stages of *in vitro* transformants. (A)** regeneration of putative transgenic shoots from cotyledonary and hypocotyl explants on 2 mg/L TDZ and 3 mg/L hygromycin medium, **(B)** multiple shoot regeneration on 2 mg/L TDZ, **(C)** rooting of transformants with elongated multiple shoots on 3 mg/L hygromycin medium, **(D)** hardening of the transformant in coco-peat, **(E)** acclimatized transgenic in the garden soil, **(F)** putative transformants growing in green house.

**Figure 3:**
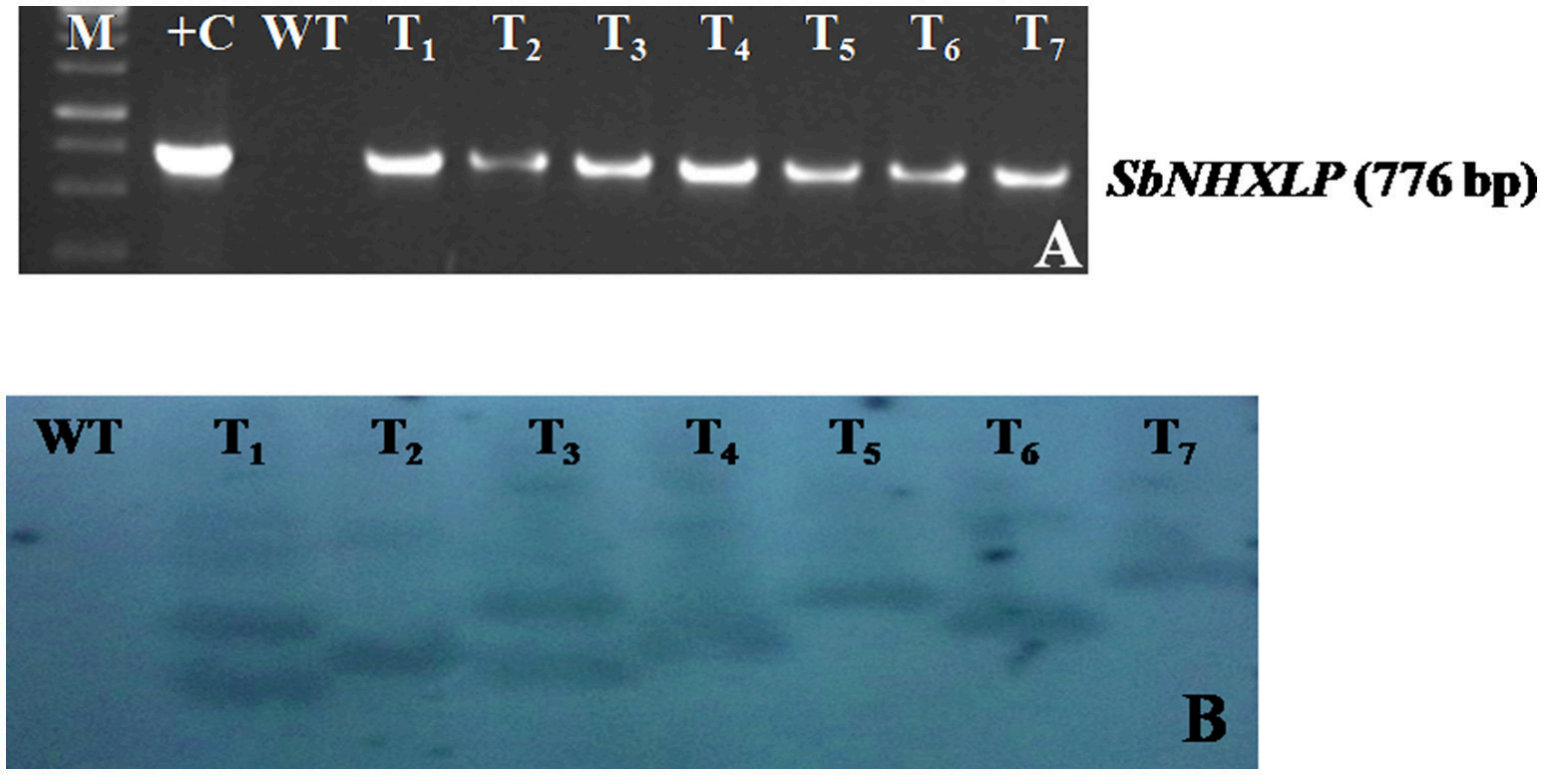
**Molecular characterization of transgenics. (A)** *SbNHXLP* PCR, **(B)** gene copy number by Southern blot analysis of genomic DNA digested with HindIII and probed with *hptII* sequence. M, molecular marker of 1 kb; +C, *SbNHXLP, Sorghum bicolor* Na^+^/H^+^ antiporter-like protein; pCAMBIA1302-*SbNHXLP* plasmid; WT, wild-type; T_1,_T_2,_T_3,_ T_4,_ T_5,_ T_6,_ and T_7_ transgenic lines.

The vector pCAMBIA1302-*SbNHXLP* was mobilized into the *A. tumefaciens* strain LBA4404 by freeze-thaw method and utilized for genetic transformation studies. Transformants from cotyledonary and hypocotyl explants were regenerated on MS medium fortified with 2 mg/L TDZ and 3 mg/L hygromycin (Figure 2A) with 44 and 32% frequencies, respectively. Transformants showed multiple shoot regeneration (Figure 2B) and rooting on 2 mg/L TDZ and 3 mg/L hygromycin (Figure 2C). After 15 days of acclimatization in coco-peat (Figure 2D), transgenics were transferred to garden soil (Figure 2E) and grown in the green house (Figure 2F). Genomic DNA was isolated from all the transgenics and plasmid DNA of pCAMBIA1302-*SbNHXLP* served as positive control. All the putative transformants showed PCR amplification (750 bp) of *SbNHXLP* (Figure 3A) and 776 bp of *hptII* (Figure S2A) genes but corresponding bands were not observed in WT. Gene copy number was confirmed by digesting the genomic DNA with *HindIII* and probed with *hptII* (Figure 3B). Four lines with single gene copies were used for further experiments. RT-PCR analysis displayed high transcript levels in the leaf tissues of transgenic lines, but not in WT (Figure S2B).

### Mendelian inheritance pattern of *hptii* in T_1_ and T_2_ generations, and subcellular localization of *Sb*NHXLP

**Figure 4:**
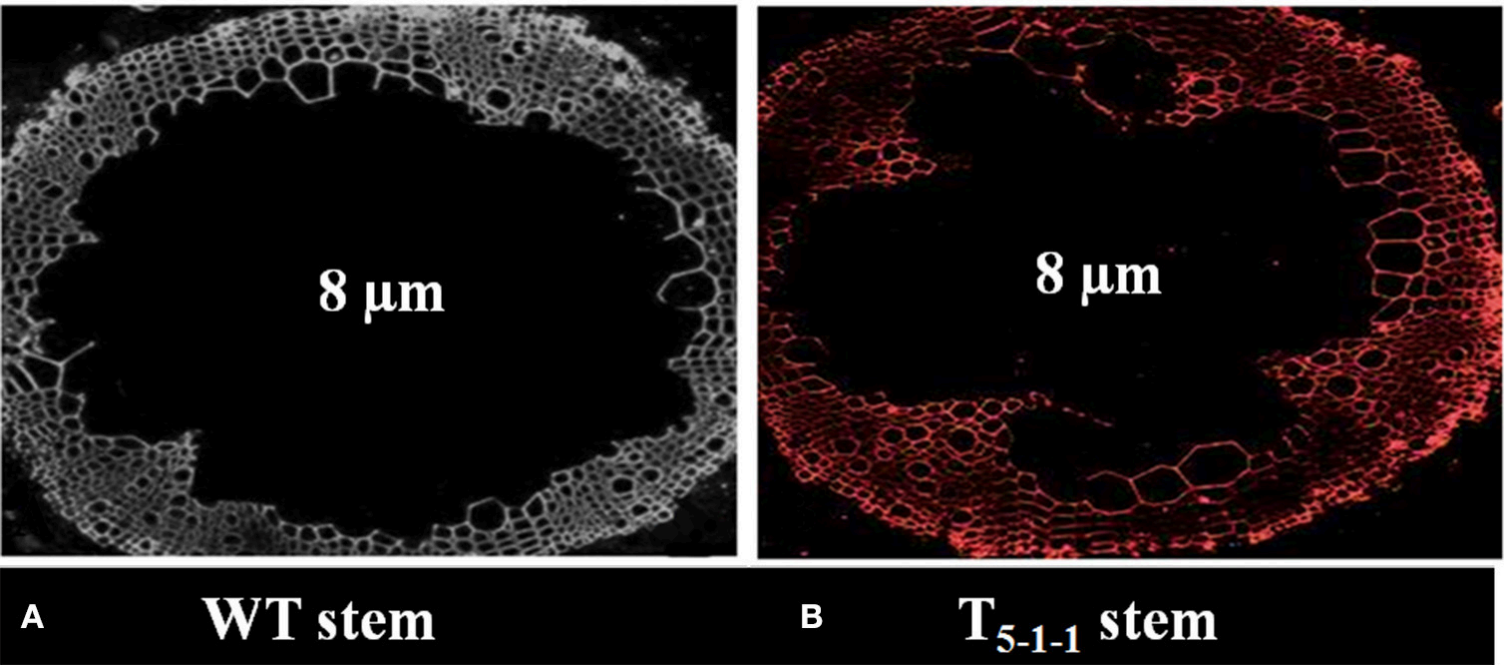
**Immunolocalization of *Sb*NHXLP protein. (A)** absence of fluorescence in WT stem, **(B)** presence of fluorescence in T_5−1−1_ stem. Fluorescence signals were emitted at the plasma membrane as revealed by using NHX antibodies conjugated with Alexafluor antibodies at 578 nm. WT, wild-type plant.

All the four T_1_ transgenic lines showed Mendelian inheritance of 3:1 (3 resistant: 1 susceptible) monogenic ratio (Figure S3 and Table S3). All T_2_ progenies segregated in 1:2:1 ratio (Figure S4 and Table S4), and the homozygous lines (T_2−1−3_,T_4−1−6_, T_5−1−1_, T_7−1−15_) were used for further analysis. At 578 nm, no fluorescence signals were noticed in WT stem, but red color fluorescence signals were emitted at the plasma membrane level in transgenic stem indicating *Sb*NHXLP localization in the membrane (Figures 4A,B).

### Overexpression of *SbNHXLP* in tomato confers salt tolerance

**Figure 5:**
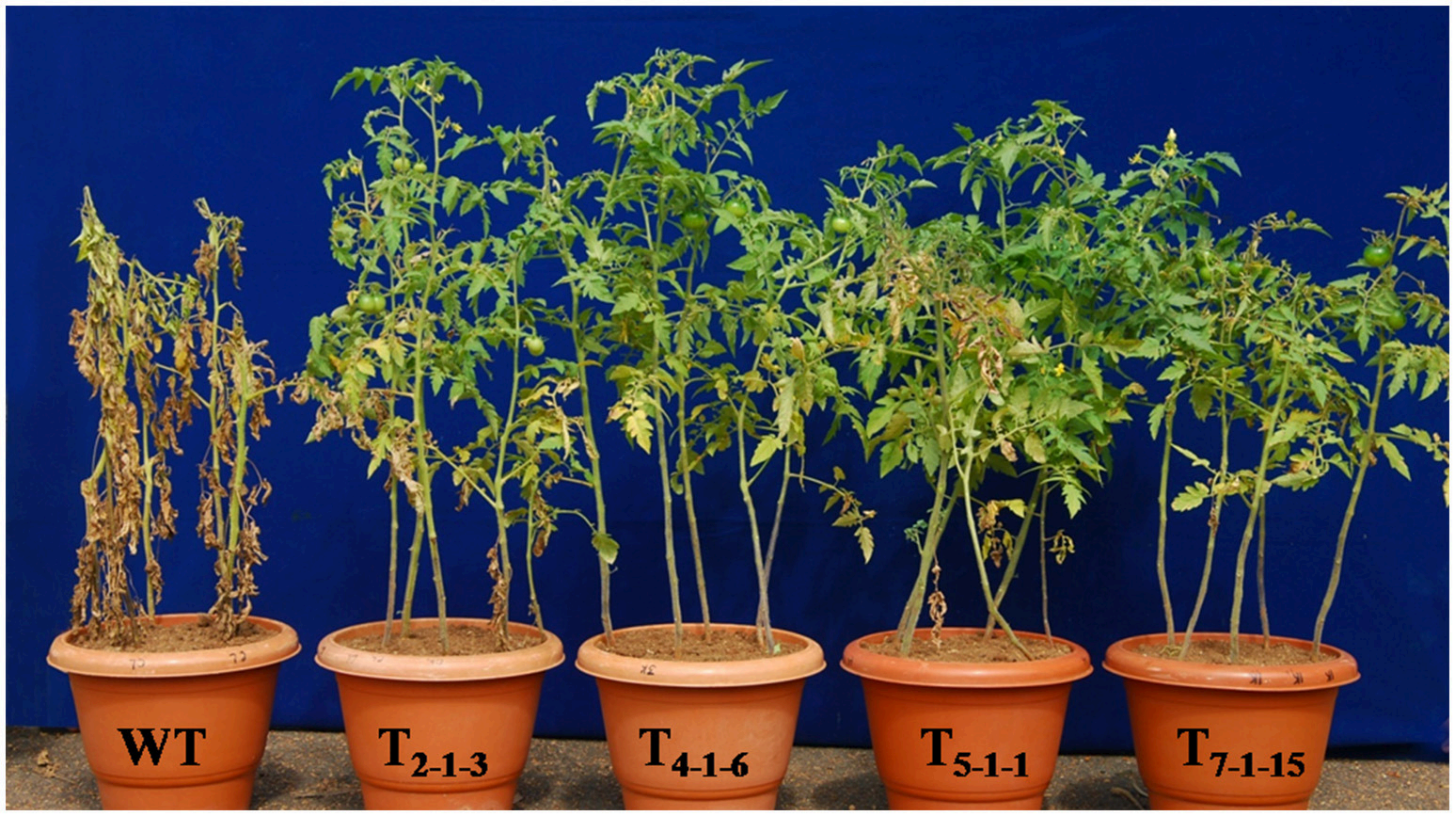
**Evaluation of transgenic tomato plants expressing *SbNHXLP* gene along with WT**. Forty-five-day-old plants were subjected to 200 mM NaCl for 15 alternate days. WT, wild-type; T_2−1−3_, T_4−1−6_, T_5−1−1_, and T_7−1−15_, transgenic lines.

Upon exposure to salt stress, WT displayed rapid leaf yellowing (including apical leaves) and turned brown. On the other hand, transgenic plants exhibited delayed leaf yellowing or partial browning after treatment. While transgenics recovered after stress treatment, WT did not and died eventually (Figure 5).

### Estimation of proline, antioxidant enzyme, and PSII activities

**Figure 6:**
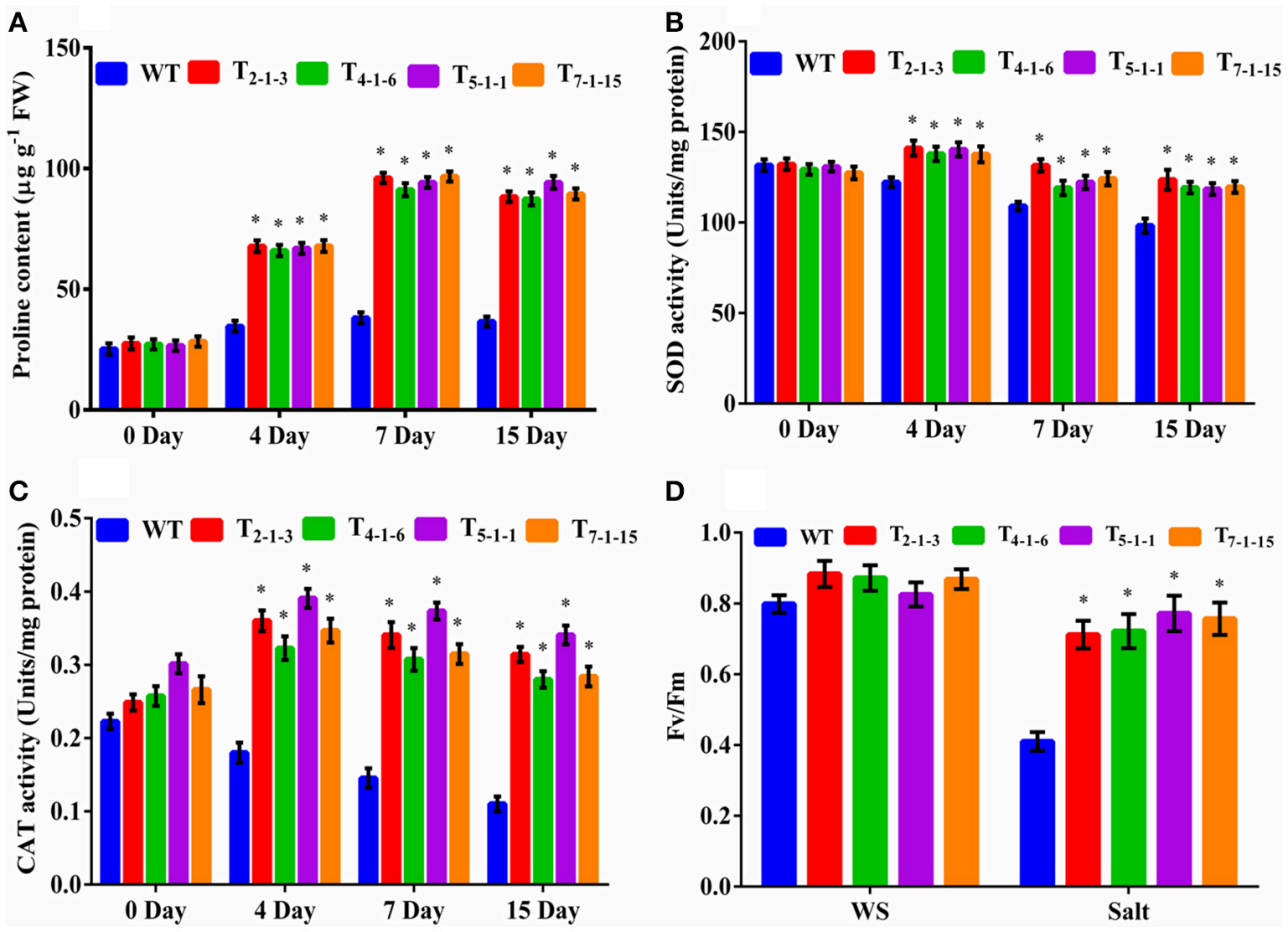
**Evaluation of transgenics under salt stresses. (A)** proline content, **(B)** SOD activity, **(C)** CAT activity, **(D)** chlorophyll fluorescence. Forty-five-day-old plants were subjected to 200 mM NaCl (salt stress) for 15 consecutive days. In each independent experiment, 5 plants were used. For chlorophyll fluorescence, 45-day-old plants were treated with 200 mM NaCl for 3 consecutive days. In each independent experiment, 2 plants were used. The mean and SE from three independent experiments are shown. ^*^Indicates significant differences in comparison with the WT at *p* < 0.05. WT, wild-type; T_2−1−3_,T_4−1−6_, T_5−1−1_, and T_7−1−15_, transgenic lines. FW, fresh weight; Fv/Fm, chlorophyll fluorescence; WS, wothout stress.

Devoid of stress, no significant accumulation of proline was observed in transgenics and WT. Under stress, accumulation of proline was significant in transgenic lines. Transgenics displayed 4-folds higher proline content after 7-days of treatment but decreased slightly by 15th day (Figure 6A). No significant change in SOD activity was recorded in transgenics and WT without stress. But, SOD activity increased by 1.5-folds in the transgenics upon exposure to NaCl stress in comparison to WT (Figure 6B). Similarly, catalase activity in transgenics under stress conditions was 2.4-folds higher as compared to WT (Figure 6C). Before salt treatment, there was no considerable change in Fv/Fm ratio between transgenic lines and WT. After treatment with 200 mM NaCl for 72 h, photochemical activity of PSII decreased by 5 to 15% in transgenics but dramatically (by 48%) in WT. Transgenic lines recorded approximately 40% higher Fv/Fm than the WT (Figure 6D).

### Inhibition of *Sb*NHXLP by amiloride, sodium, and calcium green fluorescence imaging

**Figure 7:**
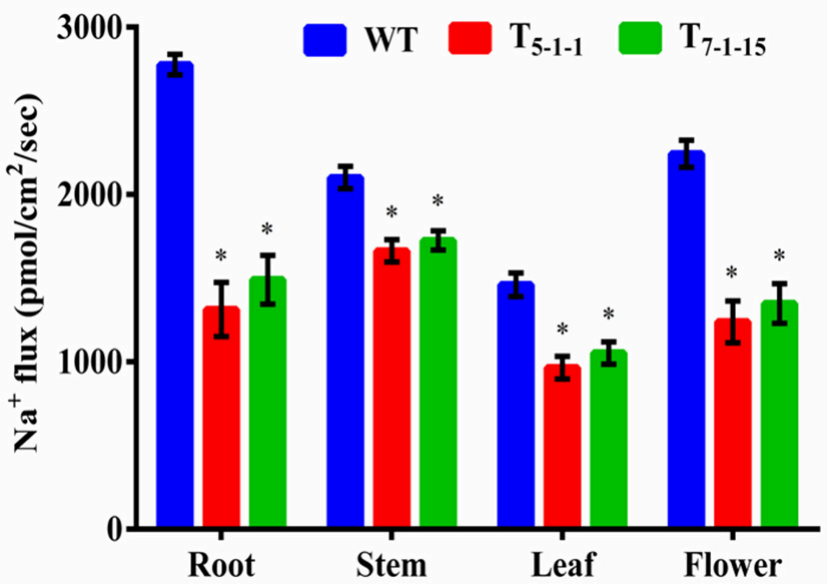
**Estimation of sodium flux by amiloride binding assay**. Forty-five-day-old transgenic and WT plants were treated with 1 mM amiloride for 2 h. In each independent experiment, 2 plants were used. The mean and SE from three independent experiments are shown. ^*^Indicates significant differences in comparison with the WT at *p* < 0.05. WT, wild-type; T_5−1−1_ and T_7−1−15,_ transgenic lines.

**Figure 8:**
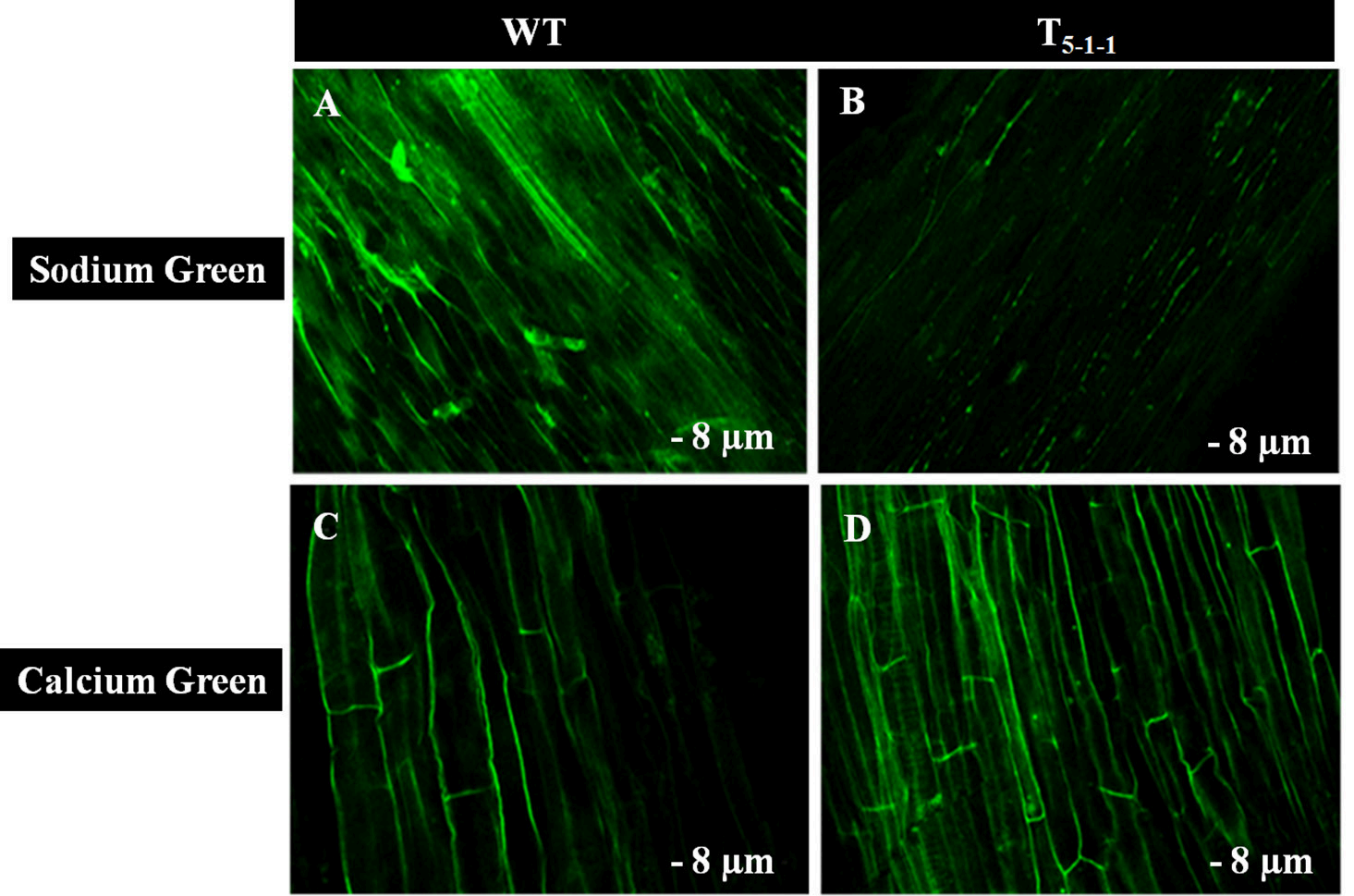
**Confocal images of Sodium Green and Calcium Green indicators in WT and T_5−1−1_ transgenic roots. (A)** Sodium Green fluorescence in WT root, **(B)** Sodium Green fluorescence in T_5−1−1_ root, **(C)** Calcium Green fluorescence in WT root, **(D)** Calcium Green fluorescence in T_5−1−1_ root. Nine-day-old seedlings were treated with 200 mM NaCl for 2 h. Root sections (8 μm) were incubated with 10 mM Sodium Green and Calcium Green solutions separately. After 1 h incubation, samples were observed under confocal microscope. WT, wild-type.

To find out whether *Sb*NHXLP excludes Na^+^ like SOS1 protein, amiloride binding assay was performed. The drug amiloride binds to the *Sb*NHXLP modeled protein motif LLFIYLLPPI, which is highly conserved among all the NHX members and inhibits the activity of these proteins. This is substantiated in transgenic plants treated with 1 mM amiloride which showed inhibition of *Sb*NHXLP activity with a decrease in Na^+^ efflux compared to WT (Figure 7). To find out if the protein is able to exclude Na^+^ and accumulate Ca^2+^ at the membrane level, roots were treated with Sodium and Calcium Green indicators. Transgenic roots showed less fluorescence indicating reduced accumulation of Na^+^ due to Na^+^ exclusion compared to WT (Figures 8A,B). Increased Calcium Green fluorescence was recorded in transgenics relative to WT (Figures 8C,D).

### Estimation of ion analysis in transgenics and WT under salt stress

**Figure 9:**
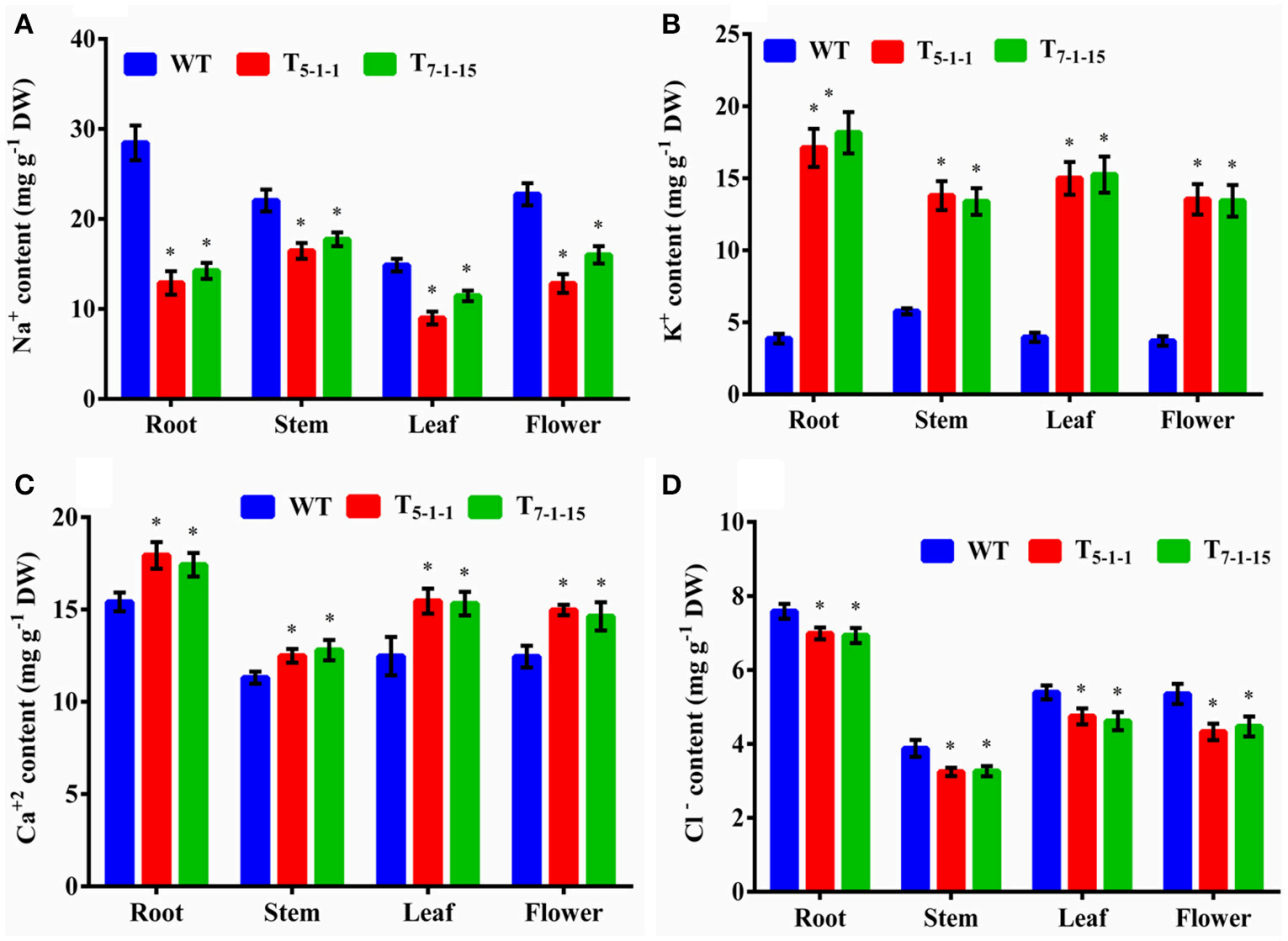
**Ion analysis of T_5−1−1_ and T_7−1−15_ transgenic lines. (A)** Na^+^, **(B)** K^+^, **(C)** Ca^2+^, **(D)** Cl^−^ content. Na^+^, K^+^, Ca^2+^, and Cl^−^ ion levels in root, stem, leaf, and flower tissues were estimated. In each independent experiment, 2 plants were used. The mean and SE from three independent experiments are shown. ^*^Indicates significant differences in comparison with the WT at *p* < 0.05. WT, wild-type; T_5−1−1_ and T_7−1−15_,transgenic lines; DW, dry weight.

Na^+^ and K^+^ homeostasis is a crucial step in plants for salt tolerance. Overexpression of *SbNHXLP* resulted in reduced Na^+^ accumulation with a concomitant increase in K^+^ under salt stress in transgenics compared to WT. At 200 mM NaCl, root, stem, leaf, and flower tissues exhibited significant reductions in Na^+^ content in T_5−1−1_ transgenic line compared to WT (Figure 9A). Transgenic leaf contained the least amount of 8.99 mg/g dry tissue, followed by root and flower. Accumulation of Na^+^ was 3-folds lower in leaf tissues in transgenics in comparison with stem. Transgenic line showed higher K^+^ content in roots (6-folds) and flowers (Figure 9B). On the other hand, Ca^2+^ content in transgenic did not differ much from that of WT (Figure 9C), while chloride content was slightly less in transgenics (Figure 9D).

### Effect of salt stress on the cambium and secondary xylem of stem and root

**Figure 10:**
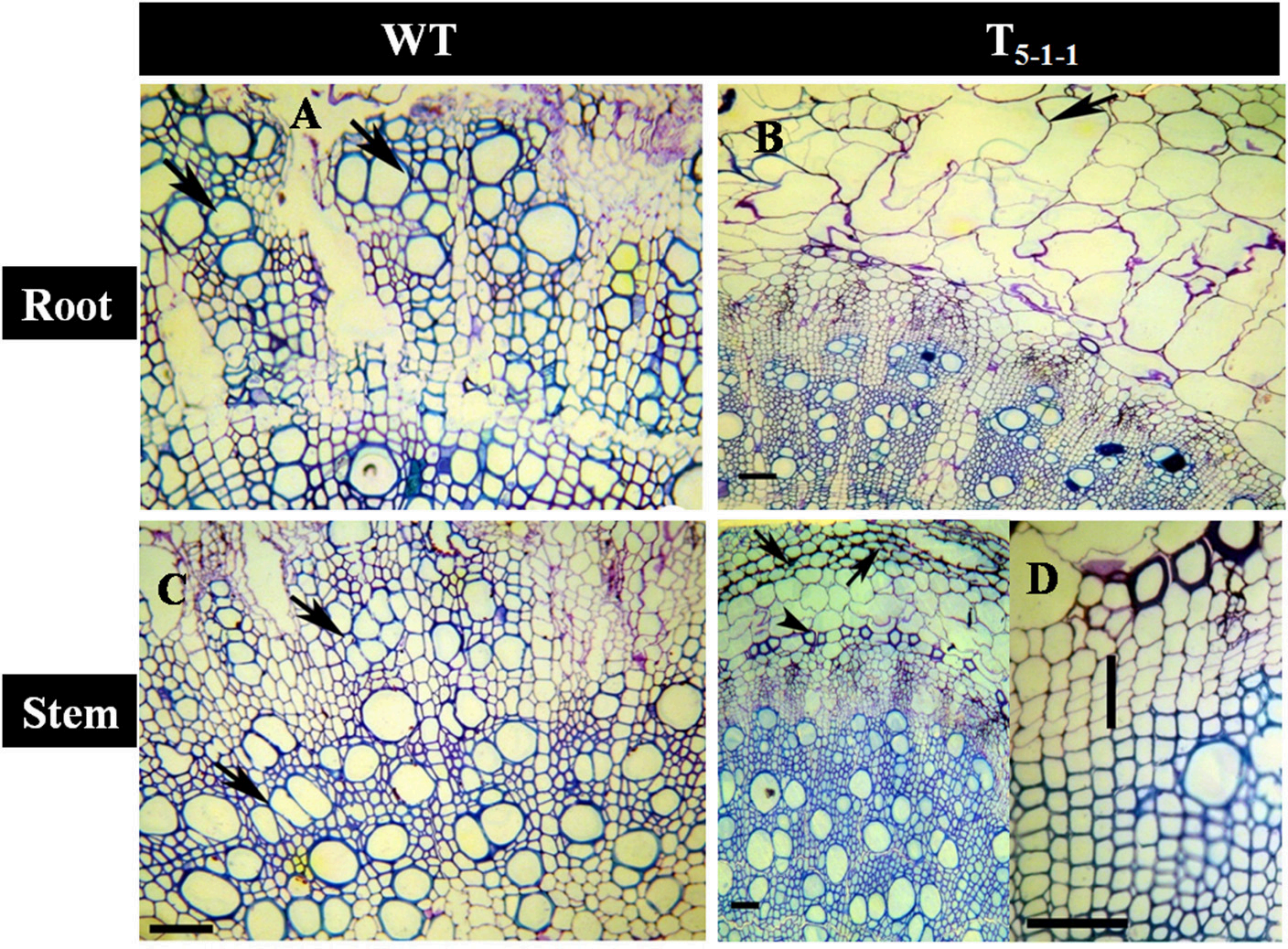
**Anatomical transverse section of T_5−1−1_ transgenic line and WT treated with 200 mM NaCl**. Transverse sections showing vascular tissues in **(A)** WT root, **(B)** T_5−1−1_ root, **(C)** WT stem, **(D)** T_5−1−1_ stem. WT root shows the occurrence of thick walled multiple vessels (arrows). In T_5−1−1_ root, radially elongated, loosely organized inner cortical cells with large intercellular spaces are seen. Arrow head indicates formation of lysigenous cavities in the cortex. WT stem shows high vessel density and thin walled fibers in the secondary xylem with the arrangement of vessels in tangential multiples (arrows). Secondary xylem of the T_5−1−1_ stem shows the distribution of solitary vessels with wide lumen and thick walled fibers. Arrows indicate the collenchymatous outer cortex. Enlarged view of T_5−1−1_ stem shows 3–4 layered cambial zones (vertical bar). Scale bar = 50 μm. WT, wild-type.

Transgenics growing under stress showed better vascular conductivity compared to WT. Transgenic root (Figure 10B) displayed multiple cambial cell layers, xylem, fiber length, width, and higher thickness. Vessel element length and width was more in transgenics compared to WT (Figure 10A). Similarly in salt treated transgenic stems, multiple cambial cell layers with increased fiber length and width, vessel length and width were noticed (Figure 10D) in comparison with WT (Figure 10C). Further, cell lysis was noticed in the outer cortex region of WT root (Figure 10A) and stem (Figure 10C). Xylogenesis was noticed in transgenic plants, as evident from the significantly higher number of cambial cell layers in the stem, amount of secondary xylem in both stem and root compared to that of salt treated WT. Dimensional changes in vascular tissues of WT and transgenics treated with salt stress are shown in Tables S5, S6, respectively.

### Fruit yield in transgenics under salt stress

Upon NaCl stress, normal flowering was noticed in transgenics, but flower drop was severe in WT. Devoid of stress, transgenics displayed reduced fruit and seed sizes when compared with WT. Without stress, the number of fruits per plant was 17.8 ± 1.18 in WT and 21.46 ± 1.06 in T_5−1−1_ transgenic line. Similarly, total fruit weight per plant was 770 ± 47.52 g in WT (without stress) and 831.8 ± 51.02 g in T_5−1−1_ transgenic line. Transgenics recovered when salt stress was relieved, but not WT, which died eventually. The number of fruits produced per plant in T_5−1−1_ transgenic line was 15.46 ± 1.23 and the total fruit weight was 582.26 ± 37.5 g after stress recovery (Table 1).

**Table 1:** **Fruit yield (number of fruits/plant and fruit weight/plant) in WT and transgenic lines**.

**Type of stress**	**Number of fruits/plant**			**Fruit weight/plant (in g)**		
	**WT**	**T_5−1−1_**	**T_7−1−15_**	**WT**	**T_5−1−1_**	**T_7−1−15_**
Without stress	17.8 ± 1.18	21.46 ± 1.06^*^	21.26 ± 1.17^*^	770.8 ± 47.52	831.8 ± 51.02	801.6 ± 61.96
200 mM NaCl	–	15.46 ± 1.23	14.4 ± 0.95	–	582.26 ± 37.5	527 ± 37.75

*Estimation of tomato fruit yield (number and weight of fruits per plant) in WT and transgenic lines. In each independent experiment, 5 plants were used. The mean and SE from three independent experiments are shown*.

* *Indicates significant differences in comparison with the WT at p < 0.05. WT, wild-type; T_5–1–1_ and T_7–1–15_, transgenic lines*.

### *Sl*CHX2-the interactant of *Sb*NHXLP protein

**Figure 11:**
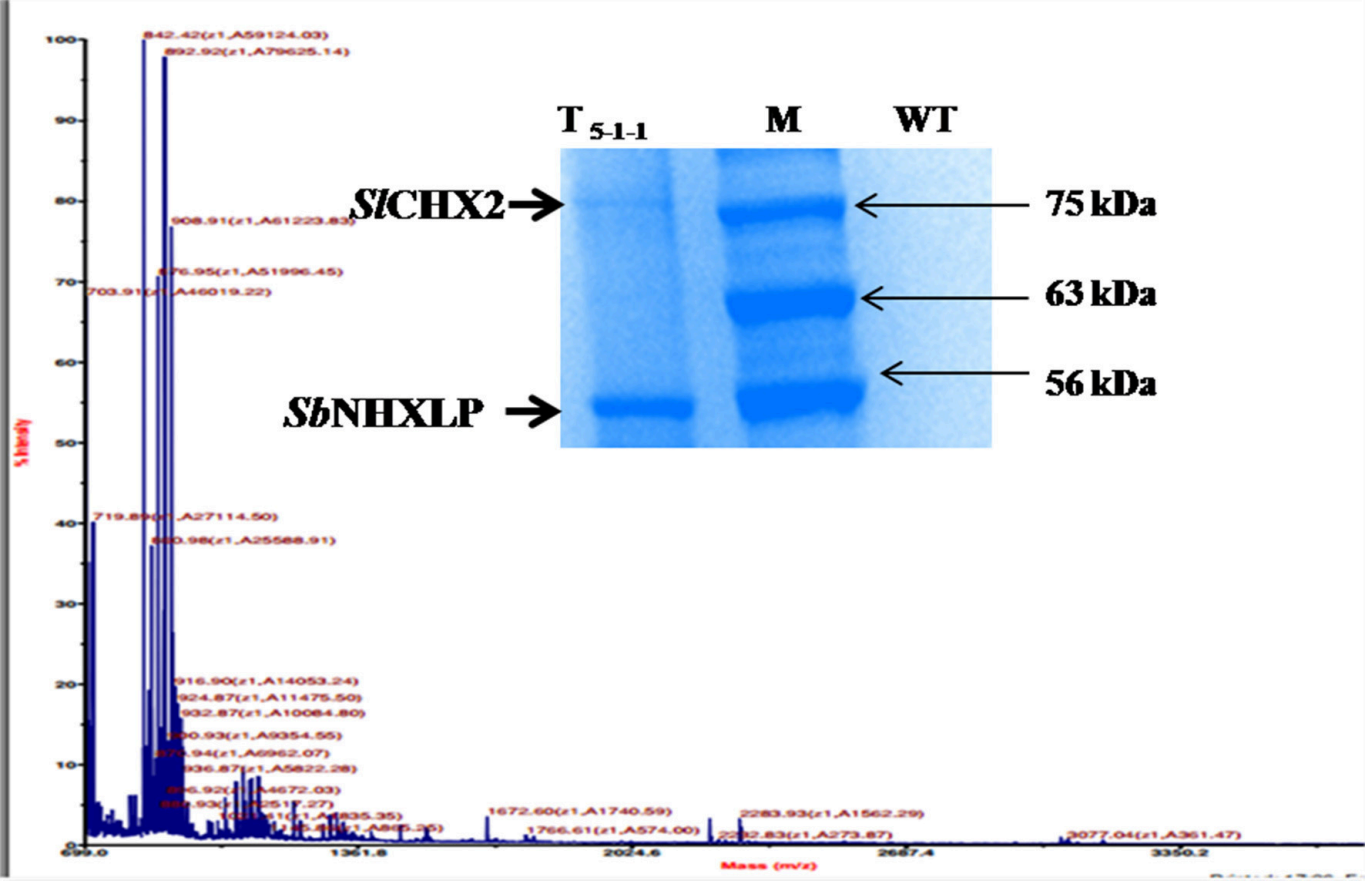
**Co-immunoprecipitation and MS-MS analysis**. MS-MS analysis of CHX2. *Sb*NHXLP-CHX2 complex was detected in immunoprecipitate of root extracts captured by anti-NHX antibody when electrophoresed with SDS-PAGE (insert).

*In silico* protein-protein interaction studies of NHX using GeneMANIA software revealed hypothetical interactions with several members of NHX and CHX families (Figure S5). Co-immunoprecipitation followed by MS-MS analysis (Figure 11) showed that *Sb*NHXLP protein interacts *in vitro* with *Solanum lycopersicum* cation proton antipoter2 (*Sl*CHX2), a member of the CPA2 family. *Sb*NHXLP-*Sl*CHX2 complex was detected in the immunoprecipitate of root extracts captured by anti-NHX antibody when electrophoresed with SDS-PAGE (Figure 11, insert) which shows that it is in agreement with *in silico* predictions. The protein was sequenced and the amino acid sequence of *Sl*CHX2 was confirmed by BLASTP analysis (Table S7).

### qRT-PCR analysis of *SbNHXLP* and *SlCHX2*

**Figure 12:**
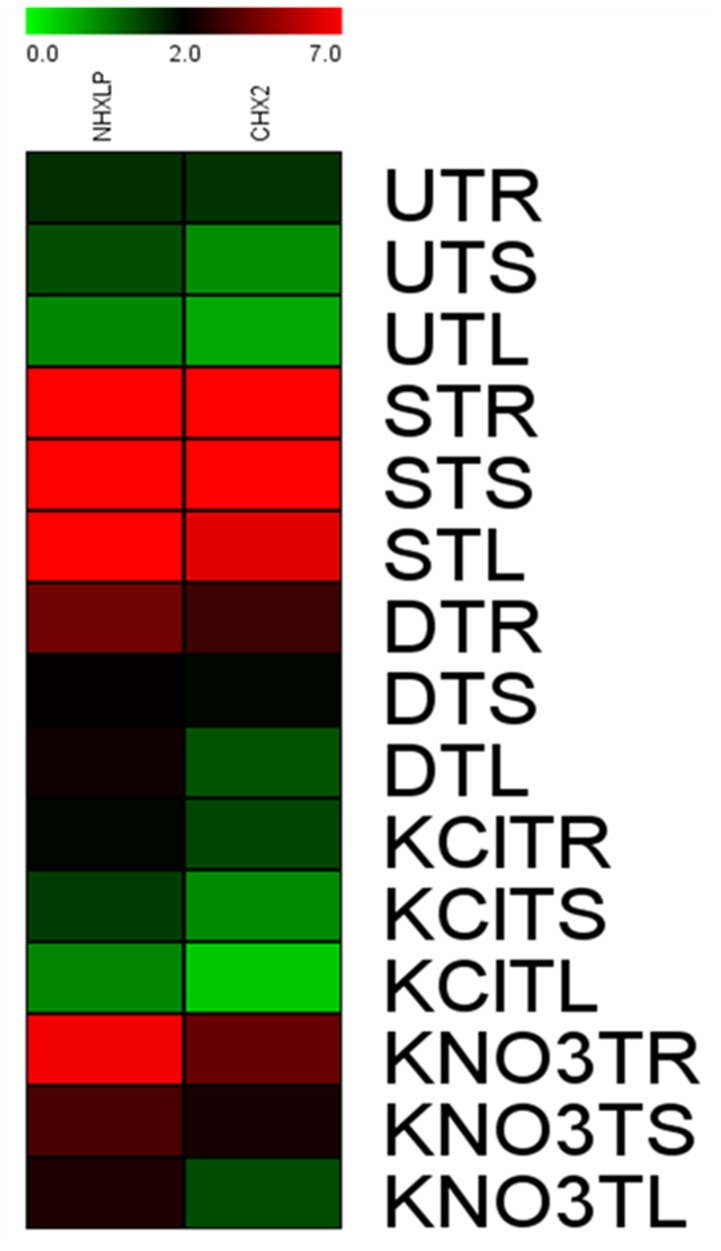
**Relative expression of *SbNHXLP*, and *SlCHX2* at the transcript level is shown in different tissues**. Relative expression of *SbNHXLP*, and *SlCHX2* are shown during different stress conditions in comparison to its control as revealed by quantitative RT-PCR analysis. Values represent the expression values obtained after normalizing against control value. All samples were analyzed in triplicates, in two independent experiments. Names on the horizontal axis indicate the genes, and the vertical axis represent the tissues, i.e., UTR, untreated transgenic root; UTS, untreated transgenic stem; UTL, untreated transgenic leaf; STR, salt-treated transgenic root; STS, salt-treated transgenic stem; STL, salt-treated transgenic leaf; DTR, drought-treated transgenic root; DTS, drought-treated transgenic stem; DTL, drought-treated transgenic leaf; KClTR, potassium chloride-treated transgenic root; KClTS, potassium chloride-treated transgenic stem; KClTL, potassium chloride-treated transgenic leaf; KNO_3_TR, potassium nitrate treated transgenic root; KNO_3_TS, potassium nitrate-treated transgenic stem; KNO_3_TL, potassium nitrate-treated transgenic leaf. Each color represents the relative expression levels. Salt, 200 mM NaCl; drought, 200 mM mannitol; KCl, 10 mM KCl, and KNO_3_, 100 mM KNO_3_.

After normalization with internal control gene β-actin, it has been observed that expression levels of *SbNHXLP* vary among the 3 tissues. *SbNHXLP* and *SlCHX2* genes displayed a differential expression in response to the stress treatments (Figure 12). Both NaCl and KNO_3_ treatments showed higher transcript abundance in root tissues of tomato for *SbNHXLP* (4.5 and 2.3-folds increase, respectively) and *SlCHX2* (4.7 and 4-folds increase, respectively). Mannitol (drought) also enhanced the activities of *SbNHXLP* (3-folds) and *SlCHX2* (1.75-folds). Contrarily, KCl treatment slightly induced the *SbNHXLP* activity (1.5-folds, in contrast to 4.5-folds under NaCl stress) but interestingly decreased the *SlCHX2* expression (1.5-folds decrease).

## Discussion

### Cloning, overexpression of *SbNHXLP* in tomato and its localization

NHX-type antiporters are pivotal players in salt tolerance and also in growth and cell expansion (Bassil et al., 2011a,b). In the present study, a member of the NHX gene family, named as *SbNHXLP* was isolated from a C_4_ cereal crop species *S. bicolor*. *Sb*NHXLP showed high homology like other NHX members (NHX2 and NHX3) at the amino acid level, but the molecular weight did not match.

Initially, tomato transformation was carried out and the results are in agreement with the earlier findings reported by Rao et al. (2009). Morphological variations in shoot or root lengths were not observed in the transgenics, but fruit and seed sizes varied when compared with WT devoid of salt stress. It has been shown that transgenics driven by constitutive promoters suffer from undesirable phenotypes, such as stunted growth and reduced yield (Wang et al., 2013). Proteins encoded by the DNA sequences of the NHX family members have been reported to be located on different cellular membranes (Zhang et al., 2015). For Na^+^ transport across the membranes, proteins encoded by members of the *NHX* family genes utilize H^+^ gradient as the driving force (Bassil et al., 2012). Our results with immunolocalization indicate that *Sb*NHXLP is a transmembrane protein localized to the plasma membrane in transgenics similar to that of SOS1 and NHX8 proteins and helps in effluxing Na^+^ (Shi et al., 2002).

### Salt tolerance in *SbNHXLP* transgenic tomatoes

Transgenics with single gene copy number were used in the present study for functional validation of the gene, since more than one copy may result in gene silencing (Tang et al., 2007). Salt stress generally causes inhibition of plant growth, reduction in photosynthesis, and protein synthesis (Hasegawa et al., 2000). Mohammad et al. (1998) and Meloni et al. (2001) observed reduction in plant height, shoot weight, and number of leaves per plant under salt stress in tomato and cotton, respectively. Contrarily, no reduction in plant height was noticed, but fruit and seed sizes decreased in the present study under salt stress. Transgenics developed with *NHX* family members conferred enhanced tolerance to salt (Shi et al., 2002; Liu et al., 2008; Rodriguez-Rosales et al., 2008; Pandey et al., 2016). In order to validate the function of *SbNHXLP*, transgenics were exposed to salt stress alongside the WT. Similar to previous reports, transgenic tomato also displayed enhanced tolerance to salt in comparison with WT. Na^+^ enters into epidermal and cortical cells through root hairs *via* non-selective cation channels. It may also enter into endodermis which does not have any casparian bands and suberin lamellae (White, 2001; Moore et al., 2002). But, plants display tolerance to NaCl stress either by excluding Na^+^ ions at the membrane level or by sequestering them into vacuoles (Shi et al., 2000; Garciadeblás et al., 2003; Apse and Blumwald, 2007), carried out by NHX family member proteins.

### Enhanced proline, antioxidant, and PSII activities in transgenics

Three to four-fold increases in proline accumulation was noticed in tomato transgenics over that of WT. Cuin and Shabala (2005) demonstrated that exogenously supplied proline significantly reduces NaCl-induced K^+^ efflux from barley roots in a dose-dependent manner. Therefore, salt-stress-induced proline accumulation may prevent NaCl-induced K^+^ leakage from the cells under salt stress conditions and thus maintain ion homeostasis. Enhanced catalase and SOD activities were noticed in transgenics indicating that *SbNHXLP* and perhaps salt-induced proline may be protecting these enzyme activities. High proline accumulating lines of niger (*Guizotia abyssinica*) displayed significantly higher antioxidant enzyme activities compared to the lines that accumulate low levels (Sarvesh et al., 1996). Proline protected antioxidative enzyme activities in transgenic sorghum plants under NaCl stress (Reddy et al., 2015). Transgenics displayed reduced photochemical PSII activity compared to WT under salt stress, indicating less chlorophyll damage in transgenics. Our observations corroborate the results obtained by Singh and Dubey (1995) and Hasegawa et al. (2000). Redondo-Gómez et al. (2007) noticed a decline in stomatal conductance resulting in reduced photosynthetic rate. But, *SbNHXLP* transgenics displayed higher Fv/Fm ratio leading to better survival under salt stress like in transgenic sorghum (Reddy et al., 2015). Thus, *SbNHXLP* appears to be protecting photosynthetic as well as antioxidative enzyme activities.

### Na^+^ exclusion at the plasma membrane and ion homeostasis in transgenics

Amiloride binds to specific signature sequence LLFIYLLPPI of NHX family members and inhibits their activity (Wu et al., 2011). Transgenic tomato overexpressing *SbNHXLP* showed reduced *Sb*NHXLP activity due to amiloride binding in comparison with WT which are not exposed to amiloride inhibition indicating that *Sb*NHXLP belongs to NHX family members. Our studies with Sodium Green dye demonstrated that transgenic root sections contained less Na^+^, compared to that of WT indicating *Sb*NHXLP is associated with Na^+^ exclusion at the plasma membrane level like SOS1 protein. Consistent with this, decreased Na^+^ levels were noticed in tomato transgenics under NaCl stress compared to WT. This is due to the overexpression of *SbNHXLP* gene, which helps in Na^+^ exclusion at the plasma membrane level. Like *SOS1, SbNHXLP* is perhaps playing a pivotal role in Na^+^ efflux being a transmembrane protein. These results indicate that plants use alternate or multiple genes for Na^+^ exclusion with redundant functions. Ion accumulation patterns point out more accumulation of Na^+^ in stems in comparison to root, flower, and leaf. It has been found out earlier that SOS1-like Na^+^/H^+^ exchanger retrieves Na^+^ from the xylem, and thus limits the rates of Na^+^ transport from the root to the shoot/leaf (Zhu et al., 2015). It appears that by retaining Na^+^ in the stems, *SbNHXLP* prevents Na^+^ from reaching leaves which are sensitive to the toxic levels of Na^+^ ions. Less accumulation of Na^+^ was also observed in other *SOS1* transgenic species like tomato (Olías et al., 2009) and tobacco (Yue et al., 2012). Concurrently, significant accumulation of K^+^ was noticed in transgenic tomato, thus balancing ion homeostasis as has also been noticed in *SOS1* transgenics (Rodriguez-Rosales et al., 2008). Calcium Green indicator displayed slightly higher calcium levels in transgenics in the present study. This is again consistent with slightly increased levels of Ca^2+^ ions in stem, leaf, and flower tissues of transgenics compared to WT.

### *SbNHXLP* overexpression improves vascular conductivity

In the present study, salt treated WT plants recorded significantly reduced secondary growth indicating that saline stress delays the process of xylogenesis in roots and stems of tomato plants. Salt treated transgenics displayed higher amount of xylem compared to that of treated WT. In soybean roots, NaCl retarded primary xylem differentiation due to delayed expression of alternate oxidase gene, but subsequently accelerated the secondary xylem differentiation (Hilal et al., 1998). In poplar, salt stress reduced the radial size of cambium and xylem differentiation has been curtailed due to diminished nutrients (Escalente-Perez et al., 2009). Enhanced xylem production in salt treated transgenic tomato plants suggests that there may not be any alternations in supply of nutrients to the cambium. Xylem in the salt exposed plants often contains vessels with small diameter than those that are grown devoid of it (Baum et al., 2000). Salt tolerant poplars showed less vessel diameter compared to salt sensitive ones (Junghans et al., 2006). We report here occurrence of multiple vessels with thicker walls in transgenics unlike that of WT. In poplar, salt stress negatively influenced the expansion of xylem vessels by decreasing biosynthesis and transport of auxin (Junghans et al., 2006). The change in vessel diameter may influence the rate of hydraulic conductivity. The large diameter vessels in the roots of *SbNHXLP* transgenics indicate a tendency for proper conductivity of water and hence better tolerance under saline conditions. A distinct change in the structure/dimensions of vessel elements and fibers to achieve more wall thickness is the salient feature found in the salt tolerant transgenics. This feature could be associated with relatively high conduit wall reinforcement that facilitates prevention of vessel collapse under osmotic stress as pointed out by Hacke and Sperry (2001).

### Enhanced fruit yield in *SbNHXLP* transgenics under stress

Devoid of salt stress, reduced fruit and seed sizes and increased number of fruits were observed in transgenics in comparison with WT. Mohammad et al. (1998) and Meloni et al. (2001) observed reduction in plant height, shoot weight, and number of leaves per plant under salt stress in tomato and cotton, respectively. It has been shown that transgenics driven by constitutive promoters suffer from undesirable phenotypes, such as stunted growth and reduced yield (Wang et al., 2013). But, how the transgene *SbNHXLP* enhances the fruit yield per plant under stress is not clearly known.

### *Sb*NHXLP-*Sl*CHX2 interaction and expression analysis

Our *in silico* analysis for PPI suggested that NHX interacts with a host of proteins, *viz*. members of NHX, CHX and our experiments with co-immunoprecipitation corroborate the same *in silico* hypothesis. Furthermore, our results on higher K^+^ accumulation in transgenic tomato plants are consistent with the assumption that *SbNHXLP* also performs the function of K^+^ acquisition like *SOS1*. *Sb*NHXLP interacts with *Sl*CHX2, a member of the CPA2 family and a putative K^+^ transporter in flowering plants (Sze et al., 2004; Chanroj et al., 2012). Mottaleb et al. (2013) found that CHX2 is localized to tonoplasts and plasma membranes and revealed that it mediates the transfer of Rb^+^ either from the vacuole to the cytosol or from the cytosol to the external medium. Since acquisition of K^+^ is inhibited under high NaCl concentrations, plants might have evolved multiple mechanisms to acquire K^+^ and to maintain ion homeostasis or high K^+^/Na^+^ ratio under these conditions by interacting with AKT1 and CHX2, which are associated with K^+^ transport. These findings suggest that *SbNHXLP* is not only involved in excluding Na^+^ from cytoplasm, but also in acquiring K^+^ and maintaining high K^+^/Na^+^ ratio in transgenics through its interaction with *Sl*CHX2. *SbNHXLP* expression was consistently higher in the root than shoot tissues and was upregulated by salt stress.

Quantitative real-time PCR analysis revealed that the expression of *SbNHXLP* and *SlCHX2* genes under NaCl and KNO_3_ treatments remained higher in root tissues than stem, and leaf tissues. Earlier, *SOS1* transcript levels have been found to be higher in *Arabidopsis* and tomato roots in response to salt stress (Shi et al., 2000; Olías et al., 2009). Similarly, Kant et al. (2006) observed increased expression of *ThSOS1* in roots compared to shoots. Activation of *CHX2* under salt stress and KNO_3_ treatments is consistent with our view that it may help to acquire more K^+^ under salt stress for maintaining proper ion homeostasis.

## Conclusion

We identified a member of the NHX gene family in *S. bicolor* and named it as *SbNHXLP* gene. It encodes a transmembrane protein. Overexpression of *SbNHXLP* in tomato plants lead to less Na^+^ and more accumulation of K^+^ in root and flower tissues indicating that it helps in ion homeostasis. Co-immunoprecipitation followed by MALDI-TOF analysis showed that *Sb*NHXLP protein interacts *in vitro* with *Sl*CHX2, belonging to CPA family and maintains ion homeostasis.

## Author contributions

PHK and PBK conceived and designed the experiments. PBK, SK, PSu, RV, and KR contributed to the experiments. PHK and PBK wrote the manuscript. RK, PSu, and RV critically analyzed the manuscript. All authors read and approved the manuscript.

## Funding

This study was supported by grants from the Department of Science and Technology (DST No: SR/SO/PS-55/07), New Delhi. PHK is thankful to the DST and UGC for providing fellowship. PBK is grateful to the CSIR, New Delhi, for providing Emeritus-fellowship.

### Conflict of interest statement

The authors declare that the research was conducted in the absence of any commercial or financial relationships that could be construed as a potential conflict of interest.

## Supplementary material

Figure S1 ***In silico* anlaysis of *SbNHXLP* gene. (A)** gene characterization, **(B)** sodium proton exchangers, **(C)** transmembrane segments, **(D)** motif analysis, and **(E)** modeling and amiloride binding (blue lines).

Figure S2 **Molecular characterization of transgenics. (A)** *hptII* PCR, **(B)** *SbNHXLP* RT-PCR. M, molecular marker of 1 kb; +C, pCAMBIA1302-*SbNHXLP* plasmid; WT, wild-type; T_1,_T_2,_T_3,_ T_4,_ T_5,_ T_6,_ and T_7_ transgenic lines.

Figure S3 **Mendelian inheritance pattern in T_1_ transgenicss along with WT seedlings on MS medium with 8 mg/L hygromycin**. WT, wild-type.

Figure S4 **Mendelian inheritance pattern in T_2_ transgenicss along with WT seedlings on MS medium with 8 mg/L hygromycin**. WT, wild-type.

Figure S5 ***In silico* protein-protein interaction of NHX proteins with CHX proteins using GeneMANIA**.

Table S1 **List of primers used in qRT-PCR**.

Table S2 **Sequences of motifs**.

Table S3 **T_1_ segregational analysis of *SbNHXLP* gene in presence of MS medium containing 8 mg/L hygromycin**. WT, wild type. χ^2^ calculated < χ^2^ tabulated 3.841 (Significance at *p* < 0.05).

Table S4 **T_2_ segregational analysis of *SbNHXLP* gene in presence of MS medium containing 8 mg/L hygromycin**. WT, wild type. χ^2^ calculated < χ^2^ tabulated 3.841 (Significance at *p* < 0.05). HR, homozygous resistant, AS, azygous sensitive.

Table S5 **Anatomical characteristics as influenced by 150 mM NaCl in the roots of WT and the transgenic line T_5−1−1_**. WT, wild type. ^*^Significant differences following ANOVA test (α = 0.05).

Table S6 **Anatomical characteristics as influenced by 150 mM NaCl in the stems of WT and the transgenic line T_5−1−1_**. WT, wild type. ^*^Significant differences following ANOVA test (α = 0.05).

Table S7 **Amino acid sequence of *Sl*CHX2 protein**.
