# Supplementary figures and images for: Overexpression of a Plasma Membrane Bound Na+/H+ Antiporter-Like Protein (SbNHXLP) Confers Salt Tolerance and Improves Fruit Yield in Tomato by Maintaining Ion Homeostasis
Source: Front Plant Sci. 2017 Jan 6;7:2027. doi: 10.3389/fpls.2016.02027 (PMC5216050; doi:10.3389/fpls.2016.02027)

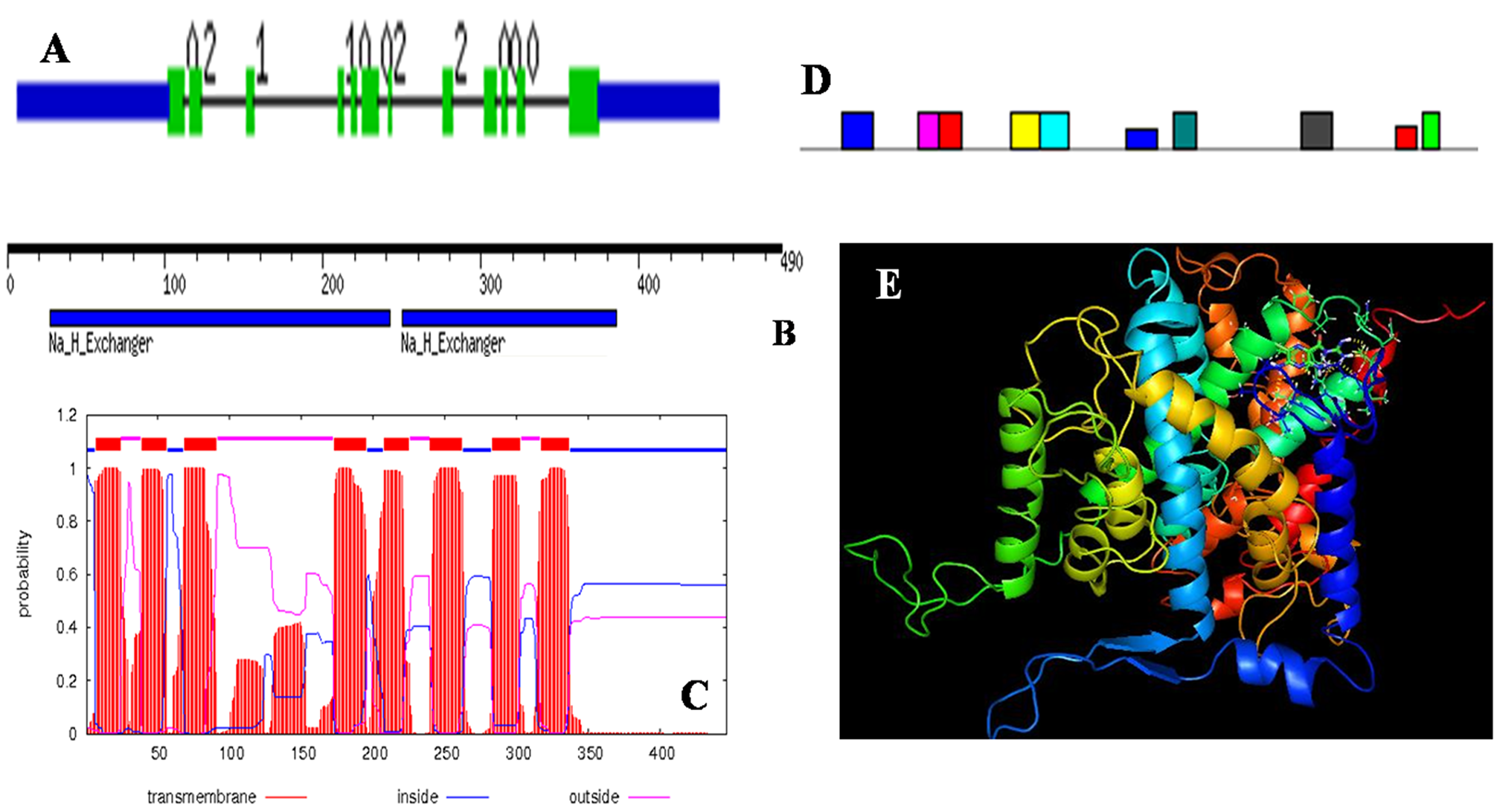

Supplement: Figure S1 — In silico anlaysis of SbNHXLP gene. (A) gene characterization, (B) sodium proton exchangers, (C) transmembrane segments, (D) motif analysis, and (E) modeling and amiloride binding (blue lines). [file Image1.TIF]

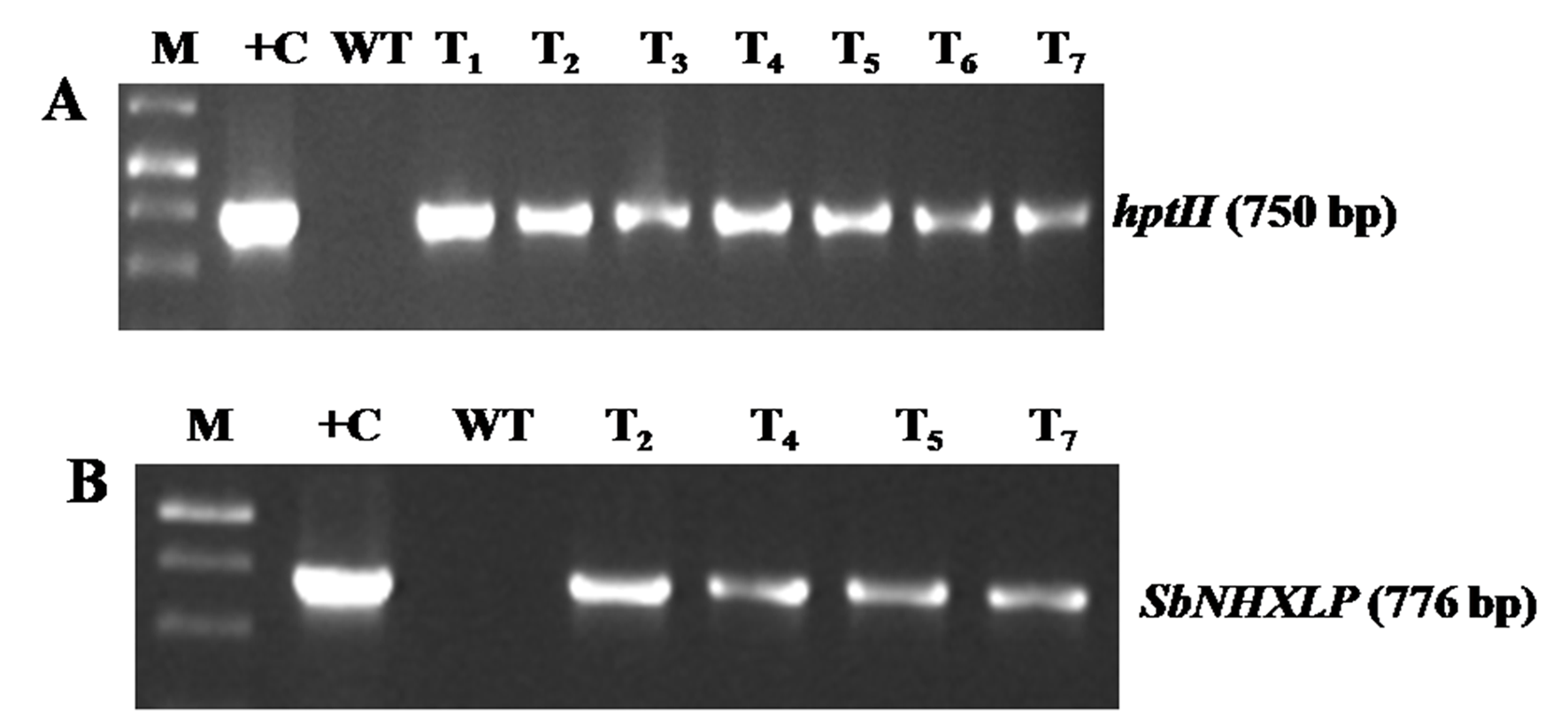

Supplement: Figure S2 — Molecular characterization of transgenics. (A) hptII PCR, (B) SbNHXLP RT-PCR. M, molecular marker of 1 kb; +C, pCAMBIA1302-SbNHXLP plasmid; WT, wild-type; T1,T2,T3, T4, T5, T6, and T7 transgenic lines. [file Image2.TIF]

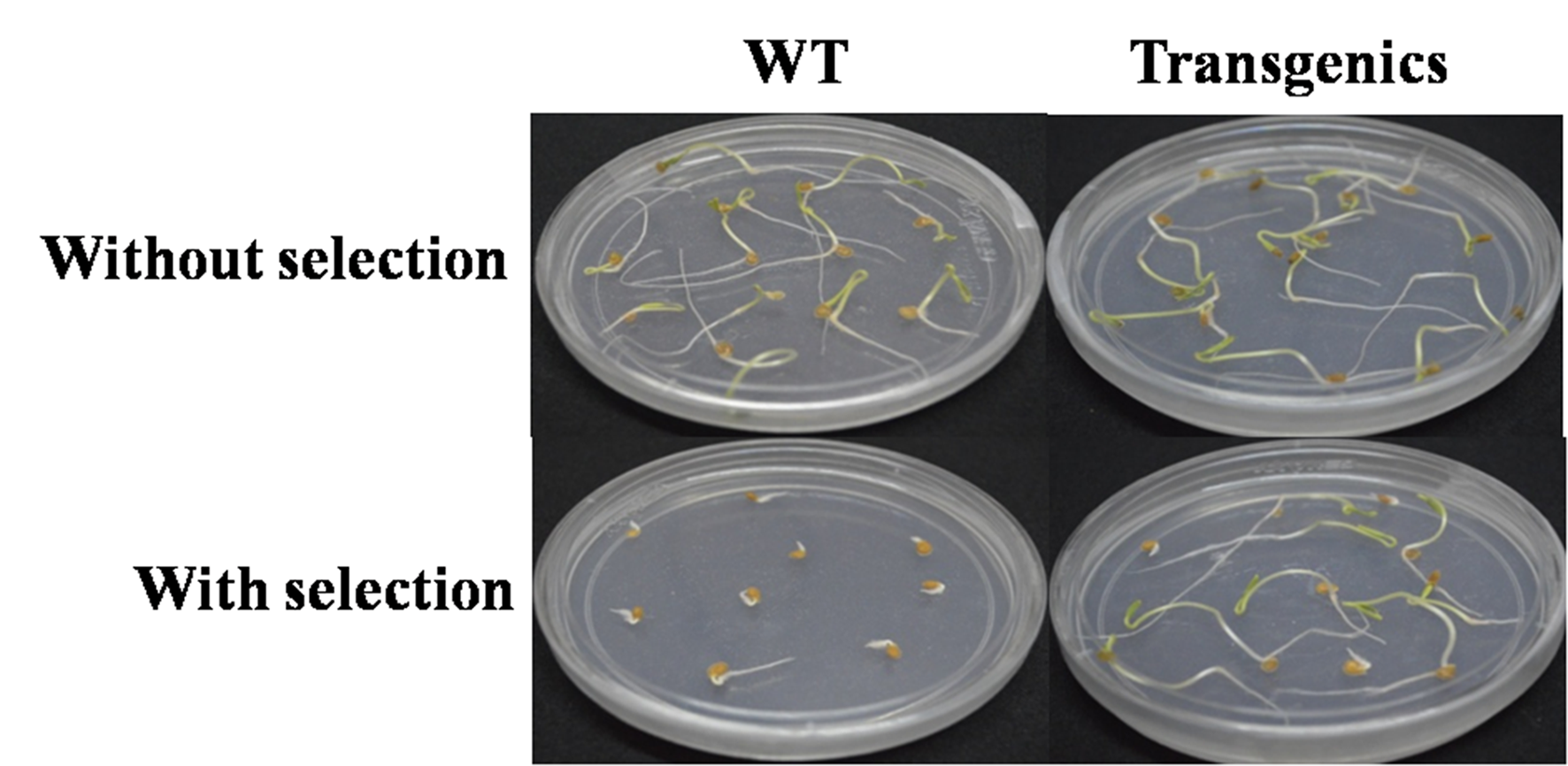

Supplement: Figure S3 — Mendelian inheritance pattern in T1 transgenicss along with WT seedlings on MS medium with 8 mg/L hygromycin. WT, wild-type. [file Image3.TIF]

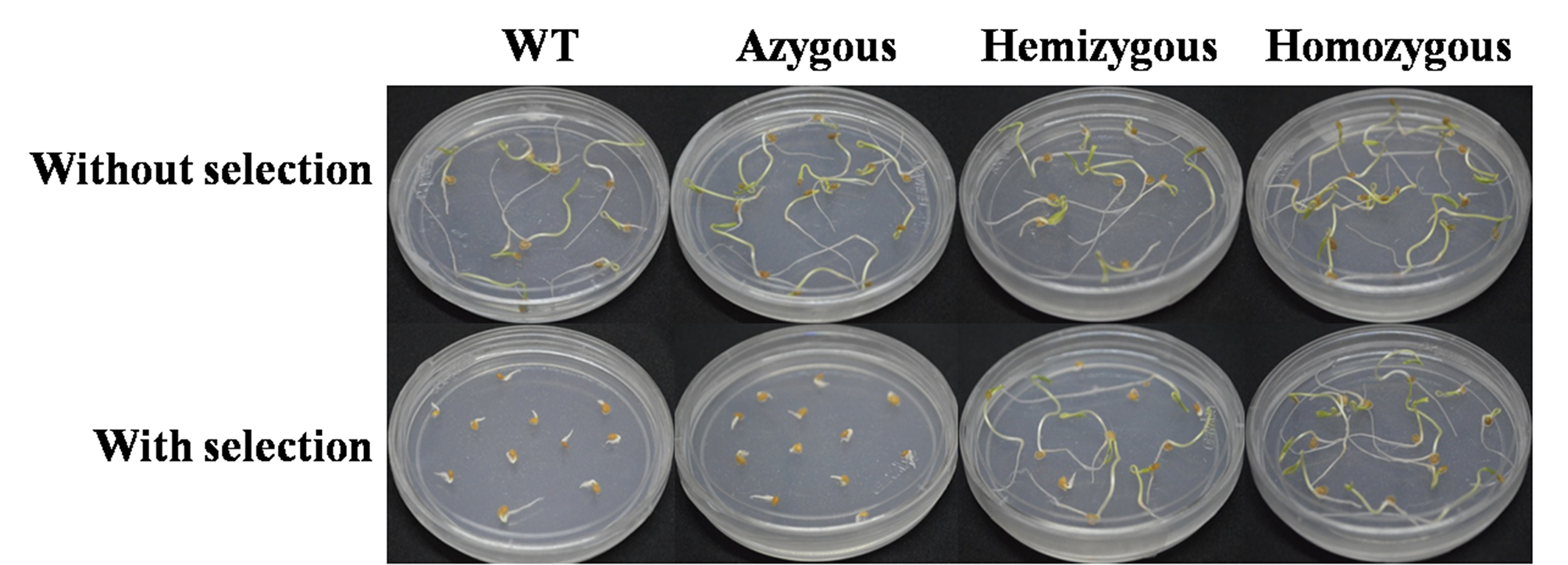

Supplement: Figure S4 — Mendelian inheritance pattern in T2 transgenicss along with WT seedlings on MS medium with 8 mg/L hygromycin. WT, wild-type. [file Image4.TIF]

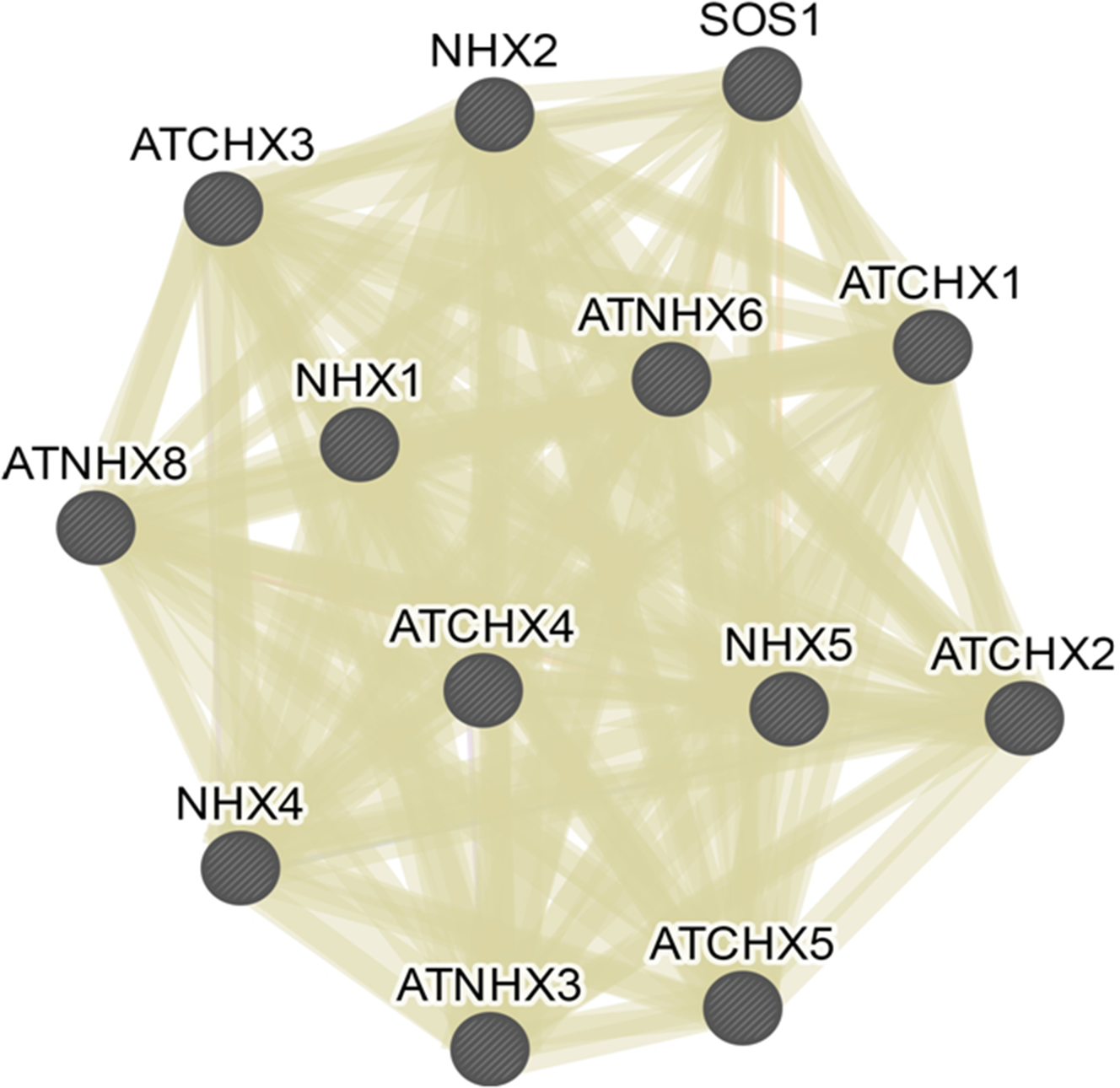

Supplement: Figure S5 — In silico protein-protein interaction of NHX proteins with CHX proteins using GeneMANIA. [file Image5.TIF]
