# Supplementary material for: Overexpression of a Plasma Membrane Bound Na+/H+ Antiporter-Like Protein (SbNHXLP) Confers Salt Tolerance and Improves Fruit Yield in Tomato by Maintaining Ion Homeostasis
Source: Front Plant Sci. 2017 Jan 6;7:2027. doi: 10.3389/fpls.2016.02027 (PMC5216050; doi:10.3389/fpls.2016.02027)
Supplement: Table S1 — List of primers used in qRT-PCR. [file Table1.DOC]

**Table S1.** List of primers used in qRT-PCR.

| **S. No.** | **Gene** | **Forward primer (5’…3’)** | **Reverse primer (5’…3’)** |
| --- | --- | --- | --- |
| 1 | *SbNHXLP* | GGTGAACGAGTCCATCACCG | GAAACCCGGCATTGAAGATTATCGG |
| 2 | *SlCHX2* | ATGGGATCTGCTGGGGATAAGTT | TTACTCTGCATCATTGGATGGATCAAC |
| 3 | *β-actin* | GCCCAAGAAATGCAAGCCGA | TAGAAGAAAGCGGTGGCCGA |
