# Supplementary material for: Overexpression of a Plasma Membrane Bound Na+/H+ Antiporter-Like Protein (SbNHXLP) Confers Salt Tolerance and Improves Fruit Yield in Tomato by Maintaining Ion Homeostasis
Source: Front Plant Sci. 2017 Jan 6;7:2027. doi: 10.3389/fpls.2016.02027 (PMC5216050; doi:10.3389/fpls.2016.02027)
Supplement: Table S2 — Sequences of motifs. [file Table2.DOC]

**Table S2.** Sequences of motifs.

| **Motif Number** | **Sequence** | **Number of amino acids** |
| --- | --- | --- |
| 1 | DETPFLYSLVFGEGVVNDA | 19 |
| 2 | AWLCACIVLGHLLEENRWVN | 20 |
| 3 | QVKKKQYFRNFMTI | 14 |
| 4 | FYIYLLPPIIFNAG | 14 |
| 5 | AIGAIFSATDSVCTLQVLNQ | 20 |
| 6 | VHYYWRKFDD | 10 |
| 7 | MDALDIEKWEFASD | 14 |
| 8 | VHGNAIMITSTITVVLFSTM | 20 |
