# Supplementary material for: Overexpression of a Plasma Membrane Bound Na+/H+ Antiporter-Like Protein (SbNHXLP) Confers Salt Tolerance and Improves Fruit Yield in Tomato by Maintaining Ion Homeostasis
Source: Front Plant Sci. 2017 Jan 6;7:2027. doi: 10.3389/fpls.2016.02027 (PMC5216050; doi:10.3389/fpls.2016.02027)
Supplement: Table S3 — T1 segregational analysis of SbNHXLP gene in presence of MS medium containing 8 mg/L hygromycin. WT, wild type. χ2 calculated < χ2 tabulated 3.841 (Significance at p < 0.05). [file Table3.DOC]

**Table S3.** T_1_ segregational analysis of *SbNHXLP* gene in presence of MS medium containing 8 mg/L hygromycin.

| T_1_ progenies | No. of seedlings tested | No. of resistant seedlings | No. of sensitive seedlings | Segregation ratio | χ^2^ | p-value |
| --- | --- | --- | --- | --- | --- | --- |
| WT | 56 | 00 | 56 | -- | -- | -- |
| T_2-1_ | 68 | 48 | 20 | 3:1 | 0.70 | 0.40 |
| T_4-1_ | 50 | 40 | 10 | 3:1 | 0.66 | 0.41 |
| T_5-1_ | 76 | 62 | 14 | 3:1 | 1.75 | 0.18 |
| T_7-1_ | 81 | 64 | 17 | 3:1 | 0.69 | 0.40 |

WT, wild type. χ^2^ calculated < χ^2^ tabulated 3.841 (Significance at p < 0.05).
