# Supplementary material for: Overexpression of a Plasma Membrane Bound Na+/H+ Antiporter-Like Protein (SbNHXLP) Confers Salt Tolerance and Improves Fruit Yield in Tomato by Maintaining Ion Homeostasis
Source: Front Plant Sci. 2017 Jan 6;7:2027. doi: 10.3389/fpls.2016.02027 (PMC5216050; doi:10.3389/fpls.2016.02027)
Supplement: Table S4 — T2 segregational analysis of SbNHXLP gene in presence of MS medium containing 8 mg/L hygromycin. WT, wild type. χ2 calculated < χ2 tabulated 3.841 (Significance at p < 0.05). HR, homozygous resistant, AS, azygous sensitive. [file Table4.DOC]

**Table S4.** T_2_ segregational analysis of *SbNHXLP* gene in presence of MS medium containing 8 mg/L hygromycin.

| T_2_ progenies | No. of seedlings tested | No. of resistant seedlings | No. of sensitive seedlings | Segregation ratio | χ^2^ | p-value |
| --- | --- | --- | --- | --- | --- | --- |
| **T_2-1_ plant progenies** | | | | | | |
| T_2-1-1_ | 60 | 50 | 10 | 3:1 | 2.22 | 0.13 |
| T_2-1-2_ | 66 | 54 | 12 | 3:1 | 1.63 | 0.20 |
| T_2-1-3_ | 70 | 70 | 00 | HR | -- | -- |
| T_2-1-4_ | 91 | 66 | 25 | 3:1 | 0.29 | 0.59 |
| T_2-1-5_ | 75 | 00 | 75 | AS | -- | -- |
| T_2-1-6_ | 48 | 48 | 00 | HR | -- | -- |
| T_2-1-7_ | 62 | 51 | 11 | 3:1 | 1.74 | 0.18 |
| T_2-1-8_ | 79 | 62 | 17 | 3:1 | 0.51 | 0.47 |
| T_2-1-9_ | 61 | 00 | 61 | AS | -- | -- |
| T_2-1-10_ | 83 | 66 | 17 | 3:1 | 0.90 | 0.34 |
| T_2-1-11_ | 59 | 59 | 00 | HR | -- | -- |
| T_2-1-12_ | 68 | 00 | 68 | AS | -- | -- |
| T_2-1-13_ | 73 | 00 | 73 | AS | -- | -- |
| T_2-1-14_ | 65 | 50 | 15 | 3:1 | 0.12 | 0.72 |
| T_2-1-15_ | 67 | 67 | 00 | HR | -- | -- |
| T_2-1-16_ | 72 | 56 | 16 | 3:1 | 0.29 | 0.59 |
| **T_4-1_ plant progenies** | | | | | | |
| T_4-1-1_ | 45 | 36 | 09 | 3:1 | 0.60 | 0.43 |
| T_4-1-2_ | 81 | 00 | 81 | AS | -- | -- |
| T_4-1-3_ | 66 | 53 | 13 | 3:1 | 0.98 | 0.32 |
| T_4-1-4_ | 88 | 69 | 19 | 3:1 | 0.54 | 0.46 |
| T_4-1-5_ | 76 | 58 | 18 | 3:1 | 0.28 | 0.59 |
| T_4-1-6_ | 55 | 55 | 00 | HR | -- | -- |
| T_4-1-7_ | 79 | 62 | 17 | 3:1 | 0.51 | 0.47 |
| T_4-1-8_ | 99 | 77 | 22 | 3:1 |  |  |
| T_4-1-9_ | 56 | 00 | 56 | AS | -- | -- |
| T_4-1-10_ | 69 | 55 | 14 | 3:1 | 0.81 | 0.36 |
| T_4-1-11_ | 73 | 59 | 14 | 3:1 | 1.31 | 0.25 |
| T_4-1-12_ | 65 | 00 | 65 | AS | -- | -- |
| T_4-1-13_ | 82 | 64 | 18 | 3:1 | 0.40 | 0.52 |
| T_4-1-14_ | 61 | 61 | 00 | HR | -- | -- |
| T_4-1-15_ | 67 | 00 | 67 | AS | -- | -- |
| T_4-1-16_ | 94 | 94 | 00 | HR | -- | -- |
| **T_5-1_ plant progenies** | | | | | | |
| T_5-1-1_ | 80 | 80 | 00 | HR | -- | -- |
| T_5-1-2_ | 78 | 63 | 15 | 3:1 | 1.38 | 0.24 |
| T_5-1-3_ | 93 | 73 | 20 | 3:1 | 0.60 | 0.43 |
| T_5-1-4_ | 66 | 00 | 66 | AS | -- | -- |
| T_5-1-5_ | 78 | 62 | 16 | 3:1 | 0.83 | 0.36 |
| T_5-1-6_ | 89 | 89 | 00 | HR | -- | -- |
| T_5-1-7_ | 54 | 46 | 08 | 3:1 | 2.98 | 0.08 |
| T_5-1-8_ | 61 | 49 | 12 | 3:1 | 0.92 | 0.33 |
| T_5-1-9_ | 76 | 76 | 00 | HR | -- | -- |
| T_5-1-10_ | 84 | 60 | 24 | 3:1 | 0.57 | 0.45 |
| T_5-1-11_ | 102 | 73 | 29 | 3:1 | 0.64 | 0.42 |
| T_5-1-12_ | 73 | 73 | 00 | HR | -- | -- |
| T_5-1-13_ | 68 | 55 | 13 | 3:1 | 1.25 | 0.26 |
| T_5-1-14_ | 52 | 00 | 52 | AS | -- | -- |
| T_5-1-15_ | 72 | 56 | 16 | 3:1 | 0.29 | 0.59 |
| T_5-1-16_ | 60 | 47 | 13 | 3:1 | 0.35 | 0.55 |
| **T_7-1_ plant progenies** | | | | | | |
| T_7-1-1_ | 80 | 58 | 22 | 3:1 | 0.26 | 0.61 |
| T_7-1-2_ | 63 | 63 | 00 | HR | -- | -- |
| T_7-1-3_ | 56 | 00 | 56 | AS | -- | -- |
| T_7-1-4_ | 88 | 69 | 19 | 3:1 | 0.54 | 0.46 |
| T_7-1-5_ | 97 | 75 | 22 | 3:1 | 0.27 | 0.60 |
| T_7-1-6_ | 49 | 00 | 49 | AS | -- | -- |
| T_7-1-7_ | 72 | 58 | 14 | 3:1 | 1.18 | 0.27 |
| T_7-1-8_ | 57 | 48 | 09 | 3:1 | 2.57 | 0.10 |
| T_7-1-9_ | 76 | 00 | 76 | AS | -- | -- |
| T_7-1-10_ | 85 | 61 | 24 | 3:1 | 0.47 | 0.49 |
| T_7-1-11_ | 69 | 55 | 14 | 3:1 | 0.81 | 0.36 |
| T_7-1-12_ | 54 | 44 | 10 | 3:1 | 1.20 | 0.27 |
| T_7-1-13_ | 67 | 49 | 18 | 3:1 | 0.12 | 0.72 |
| T_7-1-14_ | 58 | 00 | 58 | AS | -- | -- |
| T_7-1-15_ | 65 | 65 | 00 | HR | -- | -- |
| T_7-1-16_ | 73 | 73 | 00 | HR | -- | -- |
| WT | 78 | 00 | 78 | - | - | -- |

WT, wild type. χ^2^ calculated < χ^2^ tabulated 3.841 (Significance at p < 0.05). HR = homozygous resistant, AS = azygous sensitive.
