# Supplementary material for: Overexpression of a Plasma Membrane Bound Na+/H+ Antiporter-Like Protein (SbNHXLP) Confers Salt Tolerance and Improves Fruit Yield in Tomato by Maintaining Ion Homeostasis
Source: Front Plant Sci. 2017 Jan 6;7:2027. doi: 10.3389/fpls.2016.02027 (PMC5216050; doi:10.3389/fpls.2016.02027)
Supplement: Table S5 — Anatomical characteristics as influenced by 150 mM NaCl in the roots of WT and the transgenic line T5−1−1. WT, wild type. *Significant differences following ANOVA test (α = 0.05). [file Table5.DOC]

**Table S5.** Anatomical characteristics as influenced by 150 mM NaCl in the roots of WT and the transgenic line T_5-1-1_.

| **Anatomical characters** | **WT** | **T_5-1-1_** | **p-Value (WT x T_5-1-1)_** |
| --- | --- | --- | --- |
| Number of cambial cell layers | 2.6±0.5 | 3.2±0.6 | 0.038 |
| Radial extent of xylem | 285±94 | 537±93 | 0.005* |
| Fibre length | 662±78 | 558±64 | ˂0.001* |
| Fibre width | 19.3±2 | 19.6±2.9 | 0.798 |
| Fibre wall thickness | 2.10±0.5 | 3.05±0.41 | ˂0.001* |
| Vessel element length | 258±37 | 279±83 | 0.434 |
| Vessel element width | 45±12 | 70±16 | ˂0.001* |
| Vessel density | 16.3±4 | 14±1.7 | 0.189 |

WT, wild type. *Significant differences following ANOVA test (α = 0.05).
