# Supplementary material for: Overexpression of a Plasma Membrane Bound Na+/H+ Antiporter-Like Protein (SbNHXLP) Confers Salt Tolerance and Improves Fruit Yield in Tomato by Maintaining Ion Homeostasis
Source: Front Plant Sci. 2017 Jan 6;7:2027. doi: 10.3389/fpls.2016.02027 (PMC5216050; doi:10.3389/fpls.2016.02027)
Supplement: Table S6 — Anatomical characteristics as influenced by 150 mM NaCl in the stems of WT and the transgenic line T5−1−1. WT, wild type. *Significant differences following ANOVA test (α = 0.05). [file Table6.DOC]

**Table S6.** Anatomical characteristics as influenced by 150 mM NaCl in the stems of WT and the transgenic line T_5-1-1_.

| **Anatomical characters** | **WT** | **T_5-1-1_** | **p-Value (WT x T_5-1-1)_** |
| --- | --- | --- | --- |
| Number of cambial cell layers | 2.8±0.6 | 3.5±0.7 | 0.031* |
| Radial extent of xylem | 331±53 | 701±39 | 0.027* |
| Fibre length | 596±71 | 616±64 | 0.053 |
| Fibre width | 21.8±3 | 19.7±3 | 0.232 |
| Fibre wall thickness | 2.66±0.30 | 3.5±0.39 | ˂0.001* |
| Vessel element length | 268±51 | 280±62 | 0.005* |
| Vessel element width | 66±19 | 89±25 | 0.110 |
| Vessel density | 15±1.7 | 3.5±0.39 | 0.529 |

WT, wild type. *Significant differences following ANOVA test (α = 0.05).
