# Supplementary material for: Overexpression of a Plasma Membrane Bound Na+/H+ Antiporter-Like Protein (SbNHXLP) Confers Salt Tolerance and Improves Fruit Yield in Tomato by Maintaining Ion Homeostasis
Source: Front Plant Sci. 2017 Jan 6;7:2027. doi: 10.3389/fpls.2016.02027 (PMC5216050; doi:10.3389/fpls.2016.02027)
Supplement: Table S7 — Amino acid sequence of SlCHX2 protein. [file Table7.DOC]

**Table S7.** Amino acid sequence of *Sl*CHX2 protein.

MDPKLLLCLPQGDELFNPLNTMFIQMACILVFSQLFYLLLKPCGQAGPVAQILAGIVLSPVLLSRIPKVKEFFLQKNAADYYSFFSFALRTSFMFLIGLEVDLHFMRRNFKKAAVITLSSFVVSGLLSFASLMLFIPLFGIKEDYFTFFLVLLVTLSNTASPVVVRSIADWKLNTCEIGRLTISCALFIELTNVVLYTIIMAFISGTIILELFLFLLATVALILINMVLAPWLPKRNPKEKYLSKAETLVFFIFLLIIGITIESYDVNSSVSVFAIGIMFPRQGKTHRTLIQRLSYPIHEFVLPVYFGYIGFRFSIIALTKRFYLGIVIIVIVTIAGKFIGVISACMYLKIPKKYWLFLPTILSVKGHVGLLLLDSNYSEKKWWTTTIHDMMVAALVITTLVSGVLASFLLKTREKDFAYEKTSLESHNTNEELRILSCAYGVRHARGAISLVSALSGSRGASDPFTPLLMHLVPLPKKRKSELMYHEHDEDGGNANGDDEFGTNEGLEINDSIDSFAKDSKILIQQVKLVTQMLNMHEEICNATEDLRVSIVFLPFHKHQRIDGKTTNDGELFRQMNRNVLRHGPCSIGIFVDRNITGFQQPHGFDSVQHVATLFFGGPDDREALALCRWLANNTLIHLTVIQFVSEESKAETPVGNAMTRDNNEVFMEVLGRNQTEQETDRSFLEEFYNRFVTTGQVGFIEKLVSNGPHTLTILREIGEMYSLFVVGKSTGDCPMTVRMKDWEECPELGTVGDFLASSLDVNASVLVVQRQRHSHDSFIDD
